# Blahut–Arimoto Algorithms for Inner and Outer Bounds on Capacity Regions of Broadcast Channels †

**DOI:** 10.3390/e26030178

**Published:** 2024-02-20

**Authors:** Yanan Dou, Yanqing Liu, Xueyan Niu, Bo Bai, Wei Han, Yanlin Geng

**Affiliations:** 1 State Key Laboratory of ISN, Xidian University, Xi’an 710071, China; yanandou@stu.xidian.edu.cn; 2 IT Operation Center, Bank of China, Beijing 100094, China; lyq2017616@126.com; 3 Theory Lab, Central Research Institute, 2012 Labs, Huawei Tech. Co., Ltd., Shatin, N.T., Hong Kong SAR, China; niuxueyan3@huawei.com (X.N.); baibo8@huawei.com (B.B.); harvey.hanwei@huawei.com (W.H.)

**Keywords:** Blahut–Arimoto algorithm, broadcast channel, capacity region, superposition coding inner bound, Marton’s inner bound, UV outer bound

## Abstract

The celebrated Blahut–Arimoto algorithm computes the capacity of a discrete memoryless point-to-point channel by alternately maximizing the objective function of a maximization problem. This algorithm has been applied to degraded broadcast channels, in which the supporting hyperplanes of the capacity region are again cast as maximization problems. In this work, we consider general broadcast channels and extend this algorithm to compute inner and outer bounds on the capacity regions. Our main contributions are as follows: first, we show that the optimization problems are max–min problems and that the exchange of minimum and maximum holds; second, we design Blahut–Arimoto algorithms for the maximization part and gradient descent algorithms for the minimization part; third, we provide convergence analysis for both parts. Numerical experiments validate the effectiveness of our algorithms.

## 1. Introduction

In 1972, Cover [1] introduced the two-receiver discrete memoryless broadcast channel p(y,z|x) to model a system of downlink communication in which *X* is the sender and (Y,Z) are the receivers. In the same paper, he proposed a coding scheme which resulted in the superposition coding inner bound (SCIB). It turns out that the SCIB is indeed the capacity region for two-receiver broadcast channels in which the receivers are comparable in the following partial orders: degraded [2], less noisy [3], and more capable [3]. However, for a general broadcast channel the single-letter capacity region remains open.

To characterize the capacity region of a broadcast channel, a standard approach is to show that one inner bound matches another outer bound. Currently, the best inner bound for general broadcast channels is Marton’s inner bound (MIB) [4], while the UV outer bound (UVOB) [5] was the best outer bound until recently, when a better one called J version outer bound was proposed in [6].

The evaluation of inner and outer bounds is critical in the following aspects: (1) the evaluation of an inner bound usually results in an optimal input distribution which can help in the design of practical coding schemes; (2) the identification of the capacity region of a particular broadcast channel through the comparison of one inner bound and one outer bound relies on the evaluation of these two bounds; and (3) when claiming to establish a new bound, it is necessary to show that the new bound strictly improves on old ones through the evaluation of bounds on a particular channel.

**Remark** **1.** *This is the full version of conference papers accepted by ISIT 2022 and 2023 [7,8].*

However, this evaluation is usually difficult due to its non-convexity [9]. To alleviate this issue, there exist a number of generic optimization algorithms, such as interior point [10], active set [10], and sequential quadratic programming [11]. However, efficient algorithms should use the domain knowledge of information theory as well; from this viewpoint, we consider the Blahut–Arimoto (BA) algorithm, which is specially customized for information theory.

The original BA algorithm was independently developed by Blahut [12] and Arimoto [13] to calculate the channel capacity $C=\max_{q(x)}I\left(X;Y\right)$ for a general point-to-point channel p(y|x). The algorithm transforms the original maximization problem into an alternating maximization problem:

$$\begin{array}{c} \max_{q(x)}\sum_{x,y}q\left(x\right)p\left(y|x\right)\ln\frac{q(x|y)}{q(x)},\;\to \;\max_{q(x),Q(x|y)}\;\sum_{x,y}q\left(x\right)p\left(y|x\right)\ln\frac{Q(x|y)}{q(x)} \end{array}$$

where the updating formulae are explicit within each iteration.

There have been numerous extensions of the BA algorithm to various scenarios in information theory. For example, [14] applied the BA algorithm to compute the sum rate of the multiple-access channel. Later, using the idea from the BA algorithm, the whole capacity region of the multiple-access channel was formulated in [15] as a rank-one constrained problem and solved by relaxation methods. It is beyond the scope of this paper to list all of these references. Instead, we discuss those papers closely related to computing the bounds on capacity regions of broadcast channels.

In [16], the authors considered the capacity region of a degraded broadcast channel p(y,z|x), where receiver *Z* is a degraded version of *Y*.

In this scenario, the capacity region of the rate pairs $\left(R_{Y},R_{Z}\right)$ is known, and can be achieved by the simplified version of superposition coding. The supporting hyperplanes can be characterized as

$$\begin{array}{c} \theta R_{Y}+\left(1-\theta \right)R_{Z}=\max_{q(u,x)}\theta I\left(X;Y|U\right)+\left(1-\theta \right)I\left(U;Z\right). \end{array}$$

Using a similar idea to that of the BA algorithm, the authors designed an algorithm to alternatively maximize the objective function.

The method in [16] is directly applicable to less noisy broadcast channels, as the characterization of the capacity region is the same as that of the degraded case. However, this equivalence no longer holds for the more capable case, as this time the value of the supporting hyperplane $\theta R_{Y}+\left(1-\theta \right)R_{Z}$ is characterized as a max–min optimization problem (e.g., see Equation (14)). As a mater of fact, the supporting hyperplanes of the above-mentioned bounds, that is, SCIB, MIB, and UVOB, are all of the max–min form. The main issue is that the minimization part is inside the maximization part, which prevents the application of the BA algorithm to the whole problem.

The algorithms for calculating inner bounds and outer bounds for general broadcast channels are very limited. The authors of [17] considered MIB (see Section 3.2) and designed a BA algorithm to compute the sum rate $R_{Y}+R_{Z}$ of the simplified version, where the auxiliary random variable W=∅. The objective function,

$$\begin{array}{c} \sum_{u,v,x,y,z}q\left(u,v\right)q\left(x|u,v\right)p\left(y,z|x\right)\ln\frac{Q(u|y)Q(v|z)}{q(u,v)} \end{array}$$

is convex in q(x|u,v), which means that the maximum input *X* is a function of (U,V). Noticing this, the authors performed optimization over all fixed mappings q(x|u,v). However, discarding *W* can result in a strictly smaller sum rate [18], making it is necessary to consider the complete version of MIB.

In this paper, we seek to design BA algorithms for general broadcast channels in order to compute the following inner and outer bounds: SCIB, MIB, and UVOB. The key difference here is that the optimization problems are max–min problems, rather than only containing a maximization part. In Table 1, we provide an intuitive comparison of related references.

The notation we use is as follows. *p* denotes a fixed (conditional) probability distribution such as p(y,z|x), while *q* and *Q* are used for (conditional) probabilities that are changeable. Calligraphic letters such as S are used to denote sets. The use of square brackets in the function f[g] means that *f* is specified by the variable *g*; $\bar{\theta }$ denotes $\bar{\theta }:=1-\theta$; and unless otherwise specified, we use the natural logarithm. To make the mathematical expressions more concise, we use the following abbreviations of the Kullback–Leibler divergences:

$$\begin{array}{cc} D_{Y}\left(p||q\right):= & \sum_{y}p\left(y\right)\ln\frac{p(y)}{q(y)},\\ D_{Y|x}\left(p||q\right):= & \sum_{y}p\left(y|x\right)\ln\frac{p(y|x)}{q(y|x)},\\ D_{Y|X}\left(p||q\right):= & \sum_{x}p\left(x\right)\cdot D_{Y|x}\left(p||q\right). \end{array}$$

The organization is as follows. First, in Section 2 we introduce the necessary background on the BA algorithm and its extension in [16]. Then, in Section 3 we extend the BA algorithm to the evaluation of SCIB, MIB, and UVOB. Convergence analyses of these algorithms are presented in Section 4. Finally, in Section 5 we perform numerical experiments to validate the effectiveness and efficiency of our algorithms.

## 2. Mathematical Background of Blahut–Arimoto Algorithms

We first introduce the standard BA algorithm in Section 2.1, as we will rely on several of its properties later. Then, in Section 2.2 we discuss why the method in [16] cannot be applied to general broadcast channels.

### 2.1. Blahut–Arimoto Algorithm for Point-to-Point Channel

For a point-to-point channel p(y|x), the capacity *C* is the maximum of the mutual information $C=\max_{q(x)}I\left(X;Y\right)=\max_{q(x)}C\left(q\right)$, where

(1) $$\begin{array}{c} C\left(q\right)=H\left(X\right)-H\left(X|Y\right)=\sum_{x,y}q\left(x\right)p\left(y|x\right)\ln\frac{q(x|y)}{q(x)}. \end{array}$$

By replacing q(x|y) with a free variable Q(x|y), the BA algorithm performs alternating maximization $\max_{q,Q}C\left(q,Q\right)$, where

(2) $$\begin{array}{c} C\left(q,Q\right)=\sum_{x,y}q\left(x\right)p\left(y|x\right)\ln\frac{Q(x|y)}{q(x)}. \end{array}$$

Notice here that we abuse the notation of C(·), which should not cause confusion in general.

The above objective function can be reformulated as follows:

(3) $$\begin{array}{cc} C(q,Q) & =\sum_{x}q\left(x\right)\left(d\left[Q\right]\left(x\right)-\ln q\left(x\right)\right), \end{array}$$

(4) $$\begin{array}{cc} \mathrm{where}\;d[Q](x) & =\sum_{y}p\left(y|x\right)\ln Q\left(x|y\right). \end{array}$$

We call this the basic form. For different scenarios, BA algorithms mainly differ in the distribution q(·) and function d[Q](·).

The following theorem (see proof in Appendix A) provides the explicit formulae for the maximum *Q* given *q* (denoted as Q[q]) and maximum *q* given *Q* (denoted as q[Q]).

**Theorem** **1.** *The following properties hold for the problem $\max_{q,Q}C\left(q,Q\right)$.*

1. *Given a fixed q, C(q,Q) is concave in Q, and the maximum point Q[q] is induced by the input and the channel,*
(5) $$\begin{array}{c} Q\left[q\right]\left(x|y\right)=q\left(x|y\right)=\frac{q(x)p(y|x)}{\sum_{x^{\prime }}q\left(x^{\prime }\right)p\left(y|x^{\prime }\right)}. \end{array}$$
*Further, the function values satisfy*
(6) $$\begin{array}{cc} C(q) & =C(q,Q[q])\geq C(q,Q). \end{array}$$
2. *Given a fixed Q, C(q,Q) is concave in q, and the maximum point q[Q] is obtained by the Lagrangian,*
(7) $$\begin{array}{c} q\left[Q\right]=\frac{\exp\{d[Q](x)\}}{\sum_{x^{\prime }}\exp\left\{d\left[Q\right]\left(x^{\prime }\right)\right\}}. \end{array}$$
*Further, evaluation of the function value results in*
(8) $$\begin{array}{cc} C(q[Q],Q) & =\ln\sum_{x^{\prime }}\exp\left\{d\left[Q\right]\left(x^{\prime }\right)\right\}=d\left[Q\right]\left(x\right)-\ln q\left[Q\right]\left(x\right),\;\forall x. \end{array}$$

Starting from an initial $q_{0}\left(x\right)>0$, the BA algorithm performs alternating maximization and produces a sequence of points

$$\begin{array}{c} q_{0}\to Q_{1}\to q_{1}\to Q_{2}\to \ldots \to q_{n}\to Q_{n+1}\to \ldots \end{array}$$

where

$$\begin{array}{cc} Q_{n} & =Q[q_{n-1}],\\ q_{n} & =q[Q_{n}] \end{array}$$

according to Equations (5) and (7), respectively.

The criterion for stopping the iterations is based on the following result [12,13].

**Proposition** **1** (Proposition 1 in [13], Theorem 2 in [12]). *It is the case that $q_{*}$ maximizes C(q) if and only if the following holds for $Q[q_{*}]$ and some scalar D:*

$$\begin{array}{c} d\left[Q\left[q_{*}\right]\right]\left(x\right)-\ln q_{*}\left(x\right)\;\left\{\begin{array}{cc} =D,\; & q_{*}\left(x\right)>0,\\ \leq D,\; & q_{*}\left(x\right)=0. \end{array}\right. \end{array}$$

**Remark** **2.** *It should be mentioned that in order to avoid infinity minus infinity for those $q_{*}\left(x\right)=0$ in the above proposition, the following equivalent formulae can be used:*

(9) $$\begin{array}{cc} d[Q[q]](x)-\ln q(x)\; & =\sum_{y}p\left(y|x\right)\ln\frac{q(x|y)}{q(x)}=\sum_{y}p\left(y|x\right)\ln\frac{p(y|x)}{q(y)}. \end{array}$$

*This is not be an issue in the BA algorithm, as $q_{n}\left(x\right)>0$ according to Equation (7).*

Thus, at the end of the *n*-th step, if the difference

$$\begin{array}{cc} \; & \max_{x}\left\{d\left[Q\left[q_{n-1}\right]\right]\left(x\right)-\ln q_{n-1}\left(x\right)\right\}-C\left(q_{n},Q_{n}\right)\\ \; & =\max_{x}\left\{d\left[Q_{n}\right]\left(x\right)-\ln q_{n-1}\left(x\right)\right\}-\ln\sum_{x^{\prime }}\exp\left\{d\left[Q_{n}\right]\left(x^{\prime }\right)\right\} \end{array}$$

is small enough then the iteration is stopped.

Summarizing the above details, we arrive at the BA algorithm depicted in Algorithm 1. The convergence of the resulted sequence of $C(q_{n},Q_{n})$ is characterized in the following theorem (see proof in Appendix A).

**Theorem** **2** (Theorem 1 in [13], Theorem 3 in [12]). *If $p_{0}>0$, then the value $C(q_{n},Q_{n})$ converges monotonically from below to the capacity C.*

**Algorithm** **1:** Computing channel capacity**Input:** p(y|x), maximum iterations *N*, threshold ϵ>0;**Initialization:** $q_{0}\left(x\right)>0$, $\epsilon_{0}>\epsilon$, n=0;**while** *n<N and $\epsilon_{n}>\epsilon$* **do**  
( ( n←n+1;

$Q_{n}=Q\left[q_{n-1}\right]$ using Equation (5);

$q_{n}=q\left[Q_{n}\right]$ using Equation (7);

$C\left(q_{n},Q_{n}\right)=\ln\sum_{x}\exp\left\{d\left[Q_{n}\right]\left(x\right)\right\}$ using Equations (8) and (4); 

$\epsilon_{n}=\max_{x}\left\{d\left[Q_{n}\right]\left(x\right)-\ln q_{n-1}\left(x\right)\right\}-C\left(q_{n},Q_{n}\right)$ using Equation (4);
**end**
**Output:** $q_{n}\left(x\right)$, $Q_{n}\left(x|y\right)$, $C(q_{n},Q_{n})$

### 2.2. Blahut–Arimoto Algorithm for Degraded Broadcast Channel

In [16], the authors considered the capacity region of the degraded broadcast channel. The original objective function for the value of the supporting hyperplane $\theta R_{Y}+\bar{\theta }R_{Z}$ (where $\theta <\frac{1}{2}$) is:

(10) $$\begin{array}{cc} \; & F(q(u,x))\\ \; & =\theta I\left(X;Y|U\right)+\bar{\theta }I\left(U;Z\right)\\ \; & =\bar{\theta }\left(I\left(X;Y|U\right)+I\left(U;Z\right)\right)-\left(\bar{\theta }-\theta \right)I\left(X;Y|U\right) \end{array}$$

(11) $$\begin{array}{c} \;\;\;\;\;\;\;\;\;=\bar{\theta }\left(H\left(U,X\right)-H\left(X|Y,U\right)-H\left(U|Z\right)\right)-\left(\bar{\theta }-\theta \right)\left(H\left(Y|U\right)-H\left(Y|X\right)\right). \end{array}$$

Similar to the BA algorithm, the new objective function is

(12) $$\begin{array}{cc} F(q,Q)\; & =\bar{\theta }\cdot \sum_{u,x}q\left(u,x\right)\left(d\left[Q\right]\left(u,x\right)-\ln q\left(u,x\right)\right), \end{array}$$

where

(13) $$\begin{array}{cc} d[Q](u,x)\; & =\sum_{y,z}p\left(y,z|x\right)\left(\ln Q\left(x|y,u\right)Q\left(u|z\right)+\frac{\bar{\theta }-\theta }{\bar{\theta }}\ln\frac{Q(y|u)}{p(y|x)}\right). \end{array}$$

Then, the authors designed an extended BA algorithm that alternately maximizes F(q,Q) and analysed the convergence.

However, the above method does not generalize to allow for evaluating the capacity bounds of general broadcast channels. The main reason can be summarized in just one sentence: the minimum of the expectation is, in general, greater than the expectation of the minimum. Taking SCIB as an example, using the representation A(U,X) in Lemma 1, the supporting hyperplane is

$$\begin{array}{cc} \theta R_{Y}+\bar{\theta }R_{Z}\; & =\max_{q(u,x)}\theta I\left(X;Y|U\right)+\bar{\theta }\cdot \min\left\{I\left(U;Z\right),I\left(U;Y\right)\right\}\\ \; & =\max_{q(u,x)}(\bar{\theta }\left(H\left(U,X\right)-H\left(X|Y,U\right)+\min\left\{-H\left(U|Z\right),-H\left(U|Y\right)\right\}\right)\\ \; & \;\;-\left(\bar{\theta }-\theta \right)\left(H\left(Y|U\right)-H\left(Y|X\right)\right)). \end{array}$$

A direct extension might try to reformulate the last expression in the form of Equation (12) by letting

$$\begin{array}{cc} d[Q](u,x)\; & =\sum_{y,z}p\left(y,z|x\right)\left(\ln Q\left(x|y,u\right)+\min\left\{\ln Q\left(u|z\right),\ln Q\left(u|y\right)\right\}+\frac{\bar{\theta }-\theta }{\bar{\theta }}\ln\frac{Q(y|u)}{p(y|x)}\right), \end{array}$$

however, this is not an equivalent reformulation, as

$$\begin{array}{c} \min\left\{-H\left(U|Z\right),-H\left(U|Y\right)\right\}\geq \sum_{u,x}q\left(u,x\right)\cdot \sum_{y,z}p\left(y,z|x\right)\cdot \min\left\{\ln Q\left(u|z\right),\ln Q\left(u|y\right)\right\}. \end{array}$$

In the following section, we first use the fact that $\min\left\{f,g\right\}=\min_{\alpha \in [0,1]}\left\{\alpha f+\bar{\alpha }g\right\}$ to parameterize the minimum, then show the max–min exchanges so that we can apply the BA algorithm in the maximization part.

## 3. Blahut–Arimoto Algorithms for Capacity Bounds of Broadcast Channels

In this section, we first introduce two inner bounds and one outer bound on the capacity region of the broadcast channel. We characterize their supporting hyperplanes as max–min problems and show that the maximum and minimum can be exchanged. Then, we design BA algorithms for the maximization parts and gradient descent algorithms for the minimization parts.

### 3.1. Superposition Coding Inner Bound

The superposition coding inner bound was proposed by Cover in [1], which corresponds to region B in the following lemma (see proof in Appendix B). This region actually has three equivalent characterizations.

**Lemma** **1** (folklore). *The following regions A, B, and C are equivalent characterizations of the superposition coding inner bound:*

$$\begin{array}{cc} A & :=\underset{q(u,x)}{⋃}A\left(U,X\right):=\left\{\begin{array}{cc} R_{Y} & \leq I(X;Y|U),\\ R_{Z} & \leq \min\{I(U;Z),I(U;Y)\}, \end{array}\right.\\ B & :=\underset{q(u,x)}{⋃}B\left(U,X\right):=\left\{\begin{array}{cc} R_{Y} & \leq I(X;Y|U),\\ R_{Z} & \leq I(U;Z),\\ R_{Y}+R_{Z} & \leq I(X;Y), \end{array}\right.\\ C & :=\underset{q(u,x)}{⋃}C\left(U,X\right):=\left\{\begin{array}{cc} R_{Z} & \leq I(U;Z),\\ R_{Y}+R_{Z} & \leq \min\{I(X;Y|U)+I(U;Z),I(X;Y)\}. \end{array}\right. \end{array}$$

To characterize the supporting hyperplane $\theta R_{Y}+\bar{\theta }R_{Z}=F$, we choose to use the representation A(U,X). It is clear that

(14) $$\begin{array}{cc} F\; & =\max_{q(u,x)}\theta I\left(X;Y|U\right)+\bar{\theta }\cdot \min\left\{I\left(U;Z\right),I\left(U;Y\right)\right\}\\ \; & =\max_{q(u,x)}\theta I\left(X;Y|U\right)+\bar{\theta }\cdot \min_{\alpha \in [0,1]}\left(\alpha I\left(U;Z\right)+\bar{\alpha }I\left(U;Y\right)\right)\\ \; & =\max_{q(u,x)}\min_{\alpha \in [0,1]}\theta I\left(X;Y|U\right)+\bar{\theta }\alpha I\left(U;Z\right)+\bar{\theta }\bar{\alpha }I\left(U;Y\right). \end{array}$$

Notice here that we cannot use the BA algorithm directly, as there is a minimum inside the maximum. If we are able to swap the orders of the maximum and minimum, then we can adopt the BA algorithm in the maximization part.

To show this kind of exchange, we first introduce a Terkelsen-type min–max result in the following lemma.

**Lemma** **2** (Corollary 2 in Appendix A of [19]). *Let $\Lambda_{d}$ be the d-dimensional simplex, i.e., $\lambda_{i}\geq 0$ and $\sum_{i=1}^{d}\lambda_{i}=1$, let P be a set of probability distributions p(u), and let $T_{i}\left(p\left(u\right)\right)$, i=1,..,d be a set of functions such that the set T defined by*

$$\begin{array}{cc} T & =\{\left(a_{1},...,a_{d}\right)\in R^{d}:a_{i}\leq T_{i}\left(p\left(u\right)\right)\;\mathrm{for}\;\mathrm{some}\;p\left(u\right)\in P\} \end{array}$$

*is a convex set; then,*

$$\underset{p(u)\in P}{\sup}\min_{\lambda \in \Lambda_{d}}\sum_{i}\lambda_{i}T_{i}\left(p\left(u\right)\right)=\min_{\lambda \in \Lambda_{d}}\underset{p(u)\in P}{\sup}\sum_{i}\lambda_{i}T_{i}\left(p\left(u\right)\right).$$

With this lemma, we are to establish the following theorem (proof in Appendix B).

**Theorem** **3.** *The supporting hyperplane $\theta R_{Y}+\bar{\theta }R_{Z}=F$ of the superposition coding inner bound is as follows: if $\theta \in [\frac{1}{2},1]$, then $F=\max_{q(x)}\theta I\left(X;Y\right)$; otherwise, if $\theta \in [0,\frac{1}{2})$ then*

$$\begin{array}{cc} F=\min\{\; & \min_{\alpha \leq \frac{1-2\theta }{1-\theta }}\max_{q(x)}\bar{\theta }\bar{\alpha }I\left(X;Y\right)+\bar{\theta }\alpha I\left(X;Z\right),\\ \; & \min_{\alpha >\frac{1-2\theta }{1-\theta }}\max_{q(u,x)}\theta I\left(X;Y|U\right)+\bar{\theta }\bar{\alpha }I\left(U;Y\right)+\bar{\theta }\alpha I\left(U;Z\right)\}. \end{array}$$

*Further, it suffices to consider the cardinality size: |U|≤|X|.*

For the maximization part and the nontrivial case where $\theta \in (0,\frac{1}{2})$, following the above theorem, two types of BA algorithms can be designed according to the value of α.

When $\alpha \in (\frac{1-2\theta }{1-\theta },1]$, the original objective function is

(15) $$\begin{array}{cc} F(\alpha ,q)\; & =\theta I\left(X;Y|U\right)+\bar{\theta }\bar{\alpha }I\left(U;Y\right)+\bar{\theta }\alpha I\left(U;Z\right)\\ \; & =\bar{\theta }\left(I\left(X;Y|U\right)+\bar{\alpha }I\left(U;Y\right)+\alpha I\left(U;Z\right)\right)-\left(\bar{\theta }-\theta \right)I\left(X;Y|U\right)\\ \; & =\bar{\theta }\left(H\left(U,X\right)-H\left(X|Y,U\right)-\bar{\alpha }H\left(U|Y\right)-\alpha H\left(U|Z\right)\right) \end{array}$$

(16) $$\begin{array}{c} \;\;\;\;\;\;+\left(\bar{\theta }-\theta \right)\left(H\left(Y|X\right)-H\left(Y|U\right)\right)\\ \;\;\;\;\;=\bar{\theta }\cdot \sum_{u,x,y,z}q\left(u,x\right)p\left(y,z|x\right)\left(\ln\frac{q(x|y,u)}{q(u,x)}q{\left(u|y\right)}^{\bar{\alpha }}q{\left(u|z\right)}^{\alpha }+\frac{\bar{\theta }-\theta }{\bar{\theta }}\ln\frac{q(y|u)}{p(y|x)}\right). \end{array}$$

By replacing the conditional *q*s with free variables *Q*s, we have the new objective function

(17) $$\begin{array}{cc} F(\alpha ,q,Q) & =\bar{\theta }\cdot \sum_{u,x}q\left(u,x\right)\left(d\left[Q\right]\left(u,x\right)-\ln q\left(u,x\right)\right), \end{array}$$

where

(18) $$\begin{array}{cc} \;d[Q](u,x) & =\sum_{y,z}p\left(y,z|x\right)\left(\ln Q\left(x|y,u\right)Q{\left(u|y\right)}^{\bar{\alpha }}Q{\left(u|z\right)}^{\alpha }+\frac{\bar{\theta }-\theta }{\bar{\theta }}\ln\frac{Q(y|u)}{p(y|x)}\right). \end{array}$$

When $\alpha \in [0,\frac{1-2\theta }{1-\theta }]$, similar to Equation (4), the new objective function is

(19) $$\begin{array}{cc} F(\alpha ,q,Q) & =\bar{\theta }\cdot \sum_{x}q\left(x\right)\left(d\left[Q\right]\left(x\right)-\ln q\left(x\right)\right), \end{array}$$

where

(20) $$\begin{array}{cc} \;d[Q](x) & =\sum_{y,z}p\left(y,z|x\right)\left(\bar{\alpha }\ln Q\left(x|y\right)+\alpha \ln Q\left(x|z\right)\right). \end{array}$$

For the minimization part, it is possible to use the optimal *q* and *Q*s obtained in the maximization part to update α. Because the values of the optimal *q* and *Q*s may vary greatly when α changes, we propose changing α locally in the neighbourhood. A candidate approach is to use the gradient descent method, as follows:

(21) $$\begin{array}{c} \alpha_{k+1}=\alpha_{k}-\tau_{k}\cdot \frac{\partial }{\partial \alpha }F\left(\alpha_{k},q\right), \end{array}$$

where

(22) $$\begin{array}{c} \frac{\partial F(\alpha ,q)}{\partial \alpha }=\left\{\begin{array}{cc} \bar{\theta }\cdot \left(I\left(X;Z\right)-I\left(X;Y\right)\right), & \;\mathrm{if}\;0\leq \alpha \leq \frac{1-2\theta }{1-\theta },\\ \bar{\theta }\cdot \left(I\left(U;Z\right)-I\left(U;Y\right)\right), & \;\mathrm{if}\;\frac{1-2\theta }{1-\theta }<\alpha \leq 1. \end{array}\right. \end{array}$$

If the change in α is sufficiently small, it can be assumed that the optimization with respect to α converges and then stop the iteration.

We summarize the above procedures in Algorithm 2. Note that the updating rules for the *q* and *Q*s depend on the interval in which the value of α falls.
**Algorithm** **2:** Computing the superposition coding inner bound for $\theta \in (0,\frac{1}{2})$**Input:** p(y,z|x), maximum iterations *K*, *N*, thresholds η, ϵ>0, step size τ>0;**Initialization:** $\alpha_{0}\in \left(0,1\right)$, $q_{0}\left(u,x\right)>0$, $\eta_{\alpha }>\eta$, k=0;**while** *k<K and $\eta_{\alpha }>\eta$* **do**


initialize $\epsilon_{q}>\epsilon$, n=0;

**while** *n<N and $\epsilon_{q}>\epsilon$* **do**



n←n+1;



$Q_{n}=Q\left[q_{n-1}\right]$ using Equation (5) similarly;



$q_{n}=q\left[Q_{n}\right]$ using Equation (7) similarly;



$F\left(\alpha_{k},q_{n},Q_{n}\right)=\bar{\theta }\cdot \ln\sum_{u,x}\exp\left\{d\left[Q_{n}\right]\right\}$ using Equation (20) or (18);



$\epsilon_{q}=\bar{\theta }\cdot \max\left\{d\left[Q_{n}\right]-\ln q_{n-1}\right\}-F\left(\alpha_{k},q_{n},Q_{n}\right)$;

**end**

( k←k+1;


calculate $\alpha_{k}$ using Equations (21) and (22);

$\alpha_{k}\leftarrow \min\left\{1,\max\left\{0,\alpha_{k}\right\}\right\}$;

$\eta_{\alpha }=\left|\alpha_{k}-\alpha_{k-1}\right|$;

$q_{0}\leftarrow q_{n}$;**end****Output:** $\alpha_{k}$, $q_{n}\left(u,x\right)$, $Q_{n}$, $F(\alpha_{k-1},q_{n},Q_{n})$

**Remark** **3.** *Given $\alpha_{k}$, according to Equation *(8),

$$\begin{array}{cc} F(\alpha_{k},q_{n},Q_{n}) & =\bar{\theta }\cdot \ln\sum_{u,x}\exp\left\{d\left[Q_{n}\right]\left(u,x\right)\right\},\\ \Rightarrow \exp\{\frac{1}{\bar{\theta }}F\left(\alpha_{k},q_{n},Q_{n}\right)\} & =\sum_{u,x}\exp\left\{d\left[Q_{n}\right]\left(u,x\right)\right\}. \end{array}$$

*According to Equation *(18) *or* (20), *$\sum_{u,x}\exp\left\{d\left[Q_{n}\right]\right\}$ equals a sum of exponents, as it is a function of $\alpha_{k}$. This is a simple function; thus, we might wonder whether we can minimize $F(\alpha_{k},q_{n},Q_{n})$ to update $\alpha_{k}$. It turns out that this kind of global updating rule can result in an oscillating effect, as can be observed from Figure 1 in [7]. The main reason for this is that q[Q] depends locally on α; therefore, it is not suitable to update α globally.*

### 3.2. Marton’s Inner Bound

Marton’s inner bound [4] refers to the union over q(u,v,w,x) (such that (U,V,W)→X→(Y,Z) is Markov) of the non-negative rate pairs $\left(R_{Y},R_{Z}\right)$ satisfying

$$\begin{array}{cc} R_{Y} & \leq I(U,W;Y),\\ R_{Z} & \leq I(V,W;Z),\\ R_{Y}+R_{Z} & \leq \min\{I(W;Y),I(W;Z)\}+I(U;Y|W)+I(V;Z|W)-I(U;V|W). \end{array}$$

For general broadcast channels, this is the most well known inner bound.

In the following, we characterize the supporting hyperplane $\theta R_{Y}+\bar{\theta }R_{Z}=M$ of MIB. Because the expressions in MIB have symmetry in *Y* and *Z*, without loss of generality we can assume that $\theta \leq \bar{\theta }$, i.e., $\theta \in [0,\frac{1}{2}]$. According to [20], the supporting hyperplane is stated in the following lemma.

**Lemma** **3.** (Equations (2) and (5) in [20]). *The supporting hyperplane $\theta R_{Y}+\bar{\theta }R_{Z}=M$ of Marton’s inner bound, where $\theta \in [0,\frac{1}{2}]$, is*

$$\begin{array}{cc} M & =\max_{q(u,v,w,x)}\min_{\alpha \in [0,1]}M\left(\alpha ,q\left(u,v,w,x\right)\right)\\ \; & =\min_{\alpha \in [0,1]}\max_{q(u,v,w,x)}M\left(\alpha ,q\left(u,v,w,x\right)\right), \end{array}$$

*where*

(23) $$\begin{array}{cc} \; & M\left(\alpha ,q\right)=\left(\bar{\theta }-\alpha \theta \right)I\left(W;Z\right)+\alpha \theta I\left(W;Y\right)+\bar{\theta }I\left(V;Z|W\right)+\theta I\left(U;Y|W\right)-\theta I\left(U;V|W\right). \end{array}$$

*Further, it suffices to consider the following cardinalities: |U|, |V|≤|X|, and |W|≤|X|+4.*

To compute the value of this supporting hyperplane, we can reformulate M(α,q) as follows:

(24) $$\begin{array}{cc} M(\alpha ,q)\; & =\bar{\theta }H\left(U,V,W,X\right)-\left(\bar{\theta }-\alpha \theta \right)H\left(W|Z\right)-\alpha \theta H\left(W|Y\right)-\bar{\theta }H\left(V|W,Z\right)\\ \; & \;-\theta H\left(U|W,Y\right)-\left(\bar{\theta }-\theta \right)H\left(U|V,W\right)-\bar{\theta }H\left(X|U,V,W\right). \end{array}$$

Then, the objective function M(α,q,Q) can be expressed as

(25) $$\begin{array}{cc} M(\alpha ,q,Q) & =\bar{\theta }\sum_{u,v,w,x}q\left(u,v,w,x\right)\left(d\left[Q\right]\left(u,v,w,x\right)-\ln q\left(u,v,w,x\right)\right), \end{array}$$

where

(26) $$\begin{array}{cc} \;d[Q]\; & =\sum_{y,z}p\left(y,z|x\right)(\frac{\bar{\theta }-\alpha \theta }{\bar{\theta }}\ln Q\left(w|z\right)+\frac{\alpha \theta }{\bar{\theta }}\ln Q\left(w|y\right)+\ln Q\left(v|w,z\right)\\ \; & \;+\frac{\theta }{\bar{\theta }}\ln Q\left(u|w,y\right)+\frac{\bar{\theta }-\theta }{\bar{\theta }}\ln Q\left(u|v,w\right)+\ln Q\left(x|u,v,w\right)). \end{array}$$

For minimization over α, similar to Section 3.1, we update α along the gradient

(27) $$\begin{array}{c} \alpha_{k+1}=\alpha_{k}-\tau_{k}\cdot \frac{\partial }{\partial \alpha }M\left(\alpha_{k},q\right)=\alpha_{k}-\tau_{k}\cdot \theta \cdot \left(I\left(W;Y\right)-I\left(W;Z\right)\right). \end{array}$$

Similar to Algorithm 2, we summarize the algorithm for MIB in Algorithm 3.
**Algorithm** **3:** Computing Marton’s inner bound for $\theta \in (0,\frac{1}{2}]$**Input:** p(y,z|x), maximum iterations *K*, *N*, thresholds η, ϵ>0, step size τ>0;**Initialization:** $\alpha_{0}\in \left(0,1\right)$, $q_{0}\left(u,v,w,x\right)>0$, $\eta_{\alpha }>\eta$, k=0;**while** *k<K and $\eta_{\alpha }>\eta$* **do**


initialize $\epsilon_{q}>\epsilon$, n=0;

**while** *n<N and $\epsilon_{q}>\epsilon$* **do**



n←n+1;



$Q_{n}=Q\left[q_{n-1}\right]$ using Equation (5) similarly;



$q_{n}=q\left[Q_{n}\right]$ using Equation (7) similarly;



$M\left(\alpha_{k},q_{n},Q_{n}\right)=\bar{\theta }\cdot \ln\sum_{u,v,w,x}\exp\left\{d\left[Q_{n}\right]\right\}$ using Equation (26);



$\epsilon_{q}=\bar{\theta }\cdot \max\left\{d\left[Q_{n}\right]-\ln q_{n-1}\right\}-M\left(\alpha_{k},q_{n},Q_{n}\right)$;

**end**

k←k+1;


calculate $\alpha_{k}$ using Equation (27);

$\alpha_{k}\leftarrow \min\left\{1,\max\left\{0,\alpha_{k}\right\}\right\}$;

$\eta_{\alpha }=\left|\alpha_{k}-\alpha_{k-1}\right|$;

$q_{0}\leftarrow q_{n}$;**end****Output:** $\alpha_{k}$, $q_{n}\left(u,v,w,x\right)$, $Q_{n}$, $M(\alpha_{k-1},q_{n},Q_{n})$

### 3.3. UV Outer Bound

The UV outer bound [5] refers to the union over q(u,v,x) of non-negative rate pairs $\left(R_{Y},R_{Z}\right)$ satisfying

$$\begin{array}{cc} R_{Y} & \leq I(U;Y),\\ R_{Z} & \leq I(V;Z),\\ R_{Y}+R_{Z} & \leq I(U;Y)+I(X;Z|U),\\ R_{Y}+R_{Z} & \leq I(V;Z)+I(X;Y|V). \end{array}$$

For general broadcast channels, this was the best outer bound until [6] strictly improved upon it over an erasure Blackwell channel. The following theorem (proof in Appendix B) characterizes the supporting hyperplanes.

**Theorem** **4** (Claim 2 and Remark 1 in [21]). *The supporting hyperplane of the UV outer bound is*

(28) $$\begin{array}{cc} \theta R_{Y}+\bar{\theta }R_{Z}\; & =\min_{\alpha ,\beta }\max_{q(u,v,x)}\bar{\theta }\alpha I\left(V;Z\right)+\bar{\theta }\bar{\alpha }I\left(X;Z|U\right)+\theta \beta I\left(U;Y\right)+\theta \bar{\beta }I\left(X;Y|V\right), \end{array}$$

*where α,β∈[0,1] satisfy $\bar{\theta }\alpha +\theta \beta \geq \max\left\{\theta ,\bar{\theta }\right\}$. Further, it suffices to consider the cardinality sizes |U|,|V|≤|X|.*

The original objective function can be reformulated as

$$\begin{array}{cc} G(\alpha ,\beta ,q)\; & =\bar{\theta }\alpha \left(I\left(X;Y|V\right)+I\left(V;Z\right)\right)-\left(\bar{\theta }\alpha -\theta \bar{\beta }\right)I\left(X;Y|V\right)\\ \; & \;+\theta \beta \left(I\left(X;Z|U\right)+I\left(U;Y\right)\right)-\left(\theta \beta -\bar{\theta }\bar{\alpha }\right)I\left(X;Z|U\right). \end{array}$$

The right-hand side contains two parts, both of which are similar to Equation (10), i.e., the objective function of the degraded broadcast channel. It seems workable to apply the BA algorithm twice, however, it should be noted that these two parts are coupled by the same q(x).

Observe that the first part depends only on q(v,x), while the other depends on q(u,x). It suffices to consider the subset of distributions such that q(u,v,x)=q(x)q(v|x)q(u|x). Thus, it is natural to decouple these two parts by fixing q(x) and applying the BA algorithm separately to q(v|x) and q(u|x). After some manipulations, we have

(29) $$\begin{array}{cc} \; & G(\alpha ,\beta ,q,Q)\\ \; & =\left(\bar{\theta }\alpha +\theta \beta \right)H\left(X\right)\\ \; & \;+\bar{\theta }\alpha \left(H\left(V|X\right)-H\left(X|Y,V\right)-H\left(V|Z\right)\right)-\left(\bar{\theta }\alpha -\theta \bar{\beta }\right)\left(H\left(Y|V\right)-H\left(Y|X\right)\right) \end{array}$$

(30) $$\begin{array}{cc} \; & \;+\theta \beta \left(H\left(U|X\right)-H\left(X|Z,U\right)-H\left(U|Y\right)\right)-\left(\theta \beta -\bar{\theta }\bar{\alpha }\right)\left(H\left(Z|U\right)-H\left(Z|X\right)\right)\\ \; & =-\left(\bar{\theta }\alpha +\theta \beta \right)\sum_{x}q\left(x\right)\ln q\left(x\right)\\ \; & \;+\bar{\theta }\alpha \sum_{x,v}q\left(x\right)q\left(v|x\right)\left(d_{1}\left[Q\right]\left(v,x\right)-\ln q\left(v|x\right)\right)\\ \; & \;+\theta \beta \sum_{x,u}q\left(x\right)q\left(u|x\right)\left(d_{2}\left[Q\right]\left(u,x\right)-\ln q\left(u|x\right)\right). \end{array}$$

The functions $d_{1}$ and $d_{2}$ in the above are

(31) $$\begin{array}{cc} d_{1}\left[Q\right]\left(v,x\right) & =\sum_{y,z}p\left(y,z|x\right)\left(\ln Q(x|y,v)Q\left(v|z\right)+\frac{\bar{\theta }\alpha -\theta \bar{\beta }}{\bar{\theta }\alpha }\ln\frac{Q(y|v)}{p(y|x)}\right), \end{array}$$

(32) $$\begin{array}{cc} d_{2}\left[Q\right]\left(u,x\right) & =\sum_{y,z}p\left(y,z|x\right)\left(\ln Q(x|z,u)Q\left(u|y\right)+\frac{\theta \beta -\bar{\theta }\bar{\alpha }}{\theta \beta }\ln\frac{Q(z|u)}{p(z|x)}\right). \end{array}$$

For fixed q(x)q(v|x)q(u|x), according to Equation (5), the optimal *Q*s are induced Q[q]s. For fixed *Q*s, according to Equation (7), for each *x* we have

(33) $$\begin{array}{cc} q[Q](v|x) & =\frac{\exp\{d_{1}\left[Q\right]\left(v,x\right)\}}{\sum_{v^{\prime }}\exp\left\{d_{1}\left[Q\right]\left(v^{\prime },x\right)\right\}}, \end{array}$$

(34) $$\begin{array}{cc} q[Q](u|x) & =\frac{\exp\{d_{2}\left[Q\right]\left(u,x\right)\}}{\sum_{u^{\prime }}\exp\left\{d_{2}\left[Q\right]\left(u^{\prime },x\right)\right\}}. \end{array}$$

The value of the objective function is

(35) $$\begin{array}{cc} \; & G\left(\alpha ,\beta ,q\left(x\right),q\left[Q\right]\left(v|x\right),q\left[Q\right]\left(u|x\right),Q\right)=\left(\bar{\theta }\alpha +\theta \beta \right)\sum_{x}q\left(x\right)\left(d\left[Q\right]\left(x\right)-\ln q\left(x\right)\right), \end{array}$$

where

(36) $$\begin{array}{c} \;d\left[Q\right]\left(x\right)=\frac{\bar{\theta }\alpha }{\bar{\theta }\alpha +\theta \beta }\ln\sum_{v}\exp\left\{d_{1}\left[Q\right]\left(v,x\right)\right\}+\frac{\theta \beta }{\bar{\theta }\alpha +\theta \beta }\ln\sum_{u}\exp\left\{d_{2}\left[Q\right]\left(u,x\right)\right\}. \end{array}$$

Again, according to Equation (7) the optimal q[Q](x) and corresponding function value are

(37) $$\begin{array}{cc} q[Q](x) & =\frac{\exp\{d[Q](x)\}}{\sum_{x^{\prime }}\exp\left\{d\left[Q\right]\left(x^{\prime }\right)\right\}}, \end{array}$$

(38) $$\begin{array}{cc} G(\alpha ,\beta ,q[Q],Q) & =\left(\bar{\theta }\alpha +\theta \beta \right)\ln\sum_{x}\exp\left\{d\left[Q\right]\left(x\right)\right\}. \end{array}$$

For minimization over (α,β), similar to Section 3.1, we update (α,β) along the gradient:

(39) $$\begin{array}{c} \alpha_{k+1}=\alpha_{k}-\tau_{k}\cdot \frac{\partial }{\partial \alpha }G\left(\alpha_{k},\beta_{k},q\right)=\alpha_{k}-\tau_{k}\cdot \bar{\theta }\cdot \left(I\left(V;Z\right)-I\left(X;Z|U\right)\right), \end{array}$$

(40) $$\begin{array}{c} \beta_{k+1}=\beta_{k}-\tau_{k}\cdot \frac{\partial }{\partial \beta }G\left(\alpha_{k},\beta_{k},q\right)=\beta_{k}-\tau_{k}\cdot \theta \cdot \left(I\left(U;Y\right)-I\left(X;Y|V\right)\right). \end{array}$$

Here, it should be mentioned that (α,β) must satisfy the constraint $\bar{\theta }\alpha +\theta \beta \geq \max\left\{\theta ,\bar{\theta }\right\}$. Thus, if the resulting $\left(\alpha_{k+1},\beta_{k+1}\right)$ violate this constraint, then we need to scale $\bar{\theta }\alpha_{k+1}+\theta \beta_{k+1}$ up to be (at least) equal to $\max\{\theta ,\bar{\theta }\}$. One way to accomplish this is to use the equality to make β dependent on α, in which case the gradient descent update becomes $\alpha_{k+1}=\alpha_{k}-\tau_{k}\cdot d_{k}$, $\beta_{k+1}=\beta_{k}-\tau_{k}\cdot \left(-\bar{\theta }d_{k}/\theta \right)$, where

$$\begin{array}{c} d_{k}=\frac{\partial }{\partial \alpha }G\left(\alpha_{k},\beta_{k},q\right)-\frac{\bar{\theta }}{\theta }\cdot \frac{\partial }{\partial \beta }G\left(\alpha_{k},\beta_{k},q\right). \end{array}$$

Similar to Algorithm 2, we summarize the algorithm for UVOB in Algorithm 4.
**Algorithm** **4:** Computing the UV outer bound**Input:** p(y,z|x), maximum iterations *K*, *N*, thresholds η, ϵ>0, step size τ>0;**Initialization:** $\alpha_{0},\beta_{0}\in \left(0,1\right)$, $q_{0}\left(u,v,x\right)>0$, $\bar{\theta }\alpha_{0}+\theta \beta_{0}\geq \max\left\{\theta ,\bar{\theta }\right\}$, $\eta_{\alpha },\eta_{\beta }>\eta$,  k=0;**while** *k<K and $\max\{\eta_{\alpha },\eta_{\beta }\}>\eta$* **do**


initialize $\epsilon_{q}>\epsilon$, n=0;

**while** *n<N and $\epsilon_{q}>\epsilon$* **do**



n←n+1;



$Q_{n}=Q\left[q_{n-1}\right]$ using Equation (5) similarly;



$q_{n}=q\left[Q_{n}\right]$ using Equations (33), (34) and (37);



$G\left(\alpha_{k},\beta_{k},q_{n},Q_{n}\right)=\left(\bar{\theta }\alpha +\theta \beta \right)\cdot \ln\sum_{x}\exp\left\{d\left[Q_{n}\right]\right\}$ using Equation (36);



$\epsilon_{q}=\left(\bar{\theta }\alpha +\theta \beta \right)\cdot \max\left\{d\left[Q_{n}\right]\left(x\right)-\ln q_{n-1}\left(x\right)\right\}-G\left(\alpha_{k},\beta_{k},q_{n},Q_{n}\right)$;

**end**

k←k+1;


calculate $\alpha_{k}$ and $\beta_{k}$ using Equations (39) and (40);

$\alpha_{k}\leftarrow \min\left\{1,\max\left\{0,\alpha_{k}\right\}\right\}$;

$\beta_{k}\leftarrow \min\left\{1,\max\left\{0,\beta_{k}\right\}\right\}$;


if $\bar{\theta }\alpha_{k}+\theta \beta_{k}<\max\left\{\theta ,\bar{\theta }\right\}$, scale up to equality;

$\eta_{\alpha }=\left|\alpha_{k}-\alpha_{k-1}\right|$

$\eta_{\beta }=\left|\beta_{k}-\beta_{k-1}\right|$;

$q_{0}\leftarrow q_{n}$;**end****Output:** $\alpha_{k}$, $\beta_{k}$, $q_{n}\left(u,v,x\right)$, $Q_{n}$, $G(\alpha_{k-1},\beta_{k-1},q_{n},Q_{n})$

## 4. Convergence Analysis

Here, we aim to show that certain convergence results hold if $q_{n}$ lies in a proper convex set which contains the global maximizer $q_{*}$. For this purpose, we first introduce the first-order characterization of a concave function.

**Lemma** **4** (Lemma 3 in [22]). *Given a convex set S, a differentiable function f is concave in S if and only if, for all x,y∈S,*

(41) $$\begin{array}{c} f\left(y\right)-f\left(x\right)-{\left(y-x\right)}^{T}{\nabla f|}_{x}\leq 0. \end{array}$$

Similar to [22], we use the superlevel set to construct the convex set S. Let $S_{F}\left(\alpha ,k\right)$ be the superlevel set of the objective function F(α,q) of SCIB:

(42) $$\begin{array}{c} S_{F}\left(\alpha ,k\right):=\left\{q|F\left(\alpha ,q\right)\geq k\right\}. \end{array}$$

For a fixed *k*, it is possible for $S_{F}\left(\alpha ,k\right)$ to contain more than one connected set. For $q\in S_{F}\left(\alpha ,k\right)$, we denote the connected set that contains *q* as $T_{F}\left(\alpha ,k,q\right)$.

Similarly, for MIB and UVOB we define the corresponding (connected) superlevel sets: $S_{M}\left(\alpha ,k\right)$, $T_{M}\left(\alpha ,k,q\right)$, $S_{G}\left(\alpha ,\beta ,k\right)$, $T_{G}\left(\alpha ,\beta ,k,q\right)$. Note that *k* here should not be confused with the notation indicating the number of iterations in the algorithms.

### 4.1. Superposition Coding Inner Bound

According to Theorem 3, the expression of the objective function F(α,q) of SCIB depends on the value of α. Without loss of generality, we can consider the objective function depicted in Equation (16). An equivalent condition for F(α,q) to be concave is provided in the following lemma.

**Lemma** **5.** *Given a convex set S with a distribution q(u,x), then F(α,q) as depicted in Equation (16) is concave in S if and only if, for all $q_{1}$, $q_{2}\in S$, we have*

$$\begin{array}{c} \bar{\theta }\left(-D_{UX}+D_{X|YU}+\bar{\alpha }D_{U|Y}+\alpha D_{U|Z}\right)+\left(\bar{\theta }-\theta \right)D_{Y|U}\leq 0, \end{array}$$

*where $D_{A|B}$ denotes $D_{A|B}(q_{2}\left|\right|q_{1})$.*

The following lemma shows that $q_{n+1}$ lies in the same connected superlevel set as that of $q_{n}$. The proof (see Appendix C) is similar to that for Lemma 4 in [16].

**Lemma** **6.** *In Algorithm 2, if $q_{n}\left(u,x\right)\in S_{F}\left(\alpha ,k\right)$, then $q_{n+1}\in T_{F}\left(\alpha ,k,q_{n}\right)$.*

Fixing α and letting $q_{*}\left(u,x\right)$ be the maximizer, the following theorem states that the function values $F(\alpha ,q_{n},Q_{n})$ converge. The proof (see Appendix C) is similar to that of Theorem 2.

**Theorem** **5.** *If $q_{*},q_{0}\in T_{F}\left(\alpha ,k,\tilde{q}\right)$ for some k and $\tilde{q}$, and if F(α,q) is concave in $T_{F}\left(\alpha ,k,\tilde{q}\right)$, then the sequence $F(\alpha ,q_{n},Q_{n})$ generated by Algorithm 2 converges monotonically from below to $F(\alpha ,q_{*})$.*

The following corollary is implied by the proof of Theorem 5.

**Corollary** **1.** *If $q_{*},q_{0}\in T_{F}\left(\alpha ,k,\tilde{q}\right)$ for some k and $\tilde{q}$, and if F(α,q) is concave in $T_{F}\left(\alpha ,k,\tilde{q}\right)$, then*

$$\begin{array}{cc} \; & F\left(\alpha ,q_{*}\right)-F\left(\alpha ,q_{N},Q_{N}\right)\leq \frac{\bar{\theta }}{N}\cdot D_{UX}(q_{*}\left|\right|q_{0}). \end{array}$$

The above analyses deal with $F(\alpha ,q_{n},Q_{n})$ for a fixed α. When $\alpha_{m}$ changes to $\alpha_{m+1}$, the estimation for the one-step change in the function value is presented in the following proposition (see proof in Appendix C).

**Proposition** **2.** *Given $\alpha_{m}$, suppose that Algorithm 2 converges to the optimal variables ${\tilde{q}}_{*}$ and ${\tilde{Q}}_{*}$ such that ${\tilde{q}}_{*}=q\left[{\tilde{Q}}_{*}\right]$ and ${\tilde{Q}}_{*}=Q\left[{\tilde{q}}_{*}\right]$. Letting $\alpha_{m+1}$ be updated using Equation (22) and letting $q_{0}={\tilde{q}}_{*}$ be the initial point for the next round, we have*

$$\begin{array}{c} F\left(\alpha_{m+1},q_{1},Q_{1}\right)\approx F\left(\alpha_{m},{\tilde{q}}_{*}\right)-\frac{{\left(\alpha_{m+1}-\alpha_{m}\right)}^{2}}{\tau_{m}}. \end{array}$$

### 4.2. Marton’s Inner Bound

Next, we present the convergence results of the BA algorithm for MIB. The proofs are omitted, as they are similar to those for SCIB.

**Lemma** **7.** *Given a convex set S with a distribution q(u,v,w,x), M(α,q) as depicted in Equation (24) is concave in S if and only if, for all $q_{1}$, $q_{2}\in S$, we have*

$$\begin{array}{cc} \; & -\bar{\theta }D_{UVWX}+\left(\bar{\theta }-\alpha \theta \right)D_{W|Z}+\alpha \theta D_{W|Y}+\bar{\theta }D_{V|WZ}\\ \; & +\theta D_{U|WY}+\left(\bar{\theta }-\theta \right)D_{U|VW}+\bar{\theta }D_{X|UVW}\leq 0, \end{array}$$

*where $D_{A|B}$ denotes $D_{A|B}(q_{2}\left|\right|q_{1})$.*

**Lemma** **8.** *In Algorithm 3, if $q_{n}\left(u,v,w,x\right)\in S_{M}\left(\alpha ,k\right)$, then $q_{n+1}\in T_{M}\left(\alpha ,k,q_{n}\right)$.*

Fixing α and letting $q_{*}\left(u,v,w,x\right)$ be the maximizer, the following theorem states that the function values $M(\alpha ,q_{n},Q_{n})$ converge.

**Theorem** **6.** *If $q_{*},q_{0}\in T_{M}\left(\alpha ,k,\tilde{q}\right)$ for some k and $\tilde{q}$ and if M(α,q) is concave in $T_{M}\left(\alpha ,k,\tilde{q}\right)$, then the sequence $M(\alpha ,q_{n},Q_{n})$ generated by Algorithm 3 converges monotonically from below to $M(\alpha ,q_{*})$.*

The following corollary is implied by the proof of Theorem 6.

**Corollary** **2.** *If $q_{*},q_{0}\in T_{M}\left(\alpha ,k,\tilde{q}\right)$ for some k and $\tilde{q}$, and if M(α,q) is concave in $T_{M}\left(\alpha ,k,\tilde{q}\right)$, then*

$$\begin{array}{cc} \; & M\left(\alpha ,q_{*}\right)-M\left(\alpha ,q_{N},Q_{N}\right)\leq \frac{\bar{\theta }}{N}\cdot D_{UVWX}(q_{*}\left|\right|q_{0}). \end{array}$$

The estimation for the one-step change in the function value for MIB is presented in the following proposition.

**Proposition** **3.** *Given $\alpha_{m}$, suppose that Algorithm 3 converges to the optimal variables ${\tilde{q}}_{*}$ and ${\tilde{Q}}_{*}$ such that ${\tilde{q}}_{*}=q\left[{\tilde{Q}}_{*}\right]$ and ${\tilde{Q}}_{*}=Q\left[{\tilde{q}}_{*}\right]$. Letting $\alpha_{m+1}$ be updated using Equation (27) and letting $q_{0}={\tilde{q}}_{*}$ be the initial point for the next round, we have*

$$\begin{array}{c} M\left(\alpha_{m+1},q_{1},Q_{1}\right)\approx M\left(\alpha_{m},{\tilde{q}}_{*}\right)-\frac{{\left(\alpha_{m+1}-\alpha_{m}\right)}^{2}}{\tau_{m}}. \end{array}$$

### 4.3. UV Outer Bound

Now, we present the convergence results of of the BA algorithm for UVOB. The proofs are again omitted, as they are similar to those of SCIB.

**Lemma** **9.** *Given a convex set S of distribution q(u,v,x), G(α,β,q) as depicted in Equation (29) is concave in S if and only if, for all $q_{1}$, $q_{2}\in S$, we have*

$$\begin{array}{cc} -\left(\bar{\theta }\alpha +\theta \beta \right)D_{X}\; & +\bar{\theta }\alpha \left(-D_{V|X}+D_{X|YV}+D_{V|Z}\right)+\left(\bar{\theta }\alpha -\theta \bar{\beta }\right)D_{Y|V}\\ \; & +\theta \beta \left(-D_{U|X}+D_{X|ZU}+D_{U|Y}\right)+\left(\theta \beta -\bar{\theta }\bar{\alpha }\right)D_{Z|U}\leq 0, \end{array}$$

*where $D_{A|B}$ denotes $D_{A|B}(q_{2}\left|\right|q_{1})$.*

**Lemma** **10.** *In Algorithm 4, if $q_{n}\left(u,v,x\right)\in S_{G}\left(\alpha ,\beta ,k\right)$, then $q_{n+1}\in T_{G}\left(\alpha ,\beta ,k,q_{n}\right)$.*

Fixing (α,β) and letting $q_{*}\left(u,v,x\right)$ be the maximizer, the following theorem states that the function values $G(\alpha ,\beta ,q_{n},Q_{n})$ converge.

**Theorem** **7.** *If $q_{*},q_{0}\in T_{G}\left(\alpha ,\beta ,k,\tilde{q}\right)$ for some k and $\tilde{q}$, and if G(α,β,q) is concave in $T_{G}\left(\alpha ,\beta ,k,\tilde{q}\right)$, then the sequence $G(\alpha ,\beta ,q_{n},Q_{n})$ generated by Algorithm 4 converges monotonically from below to $G(\alpha ,\beta ,q_{*})$.*

The following corollary is implied by the proof of Theorem 7.

**Corollary** **3.** *If $q_{*},q_{0}\in T_{G}\left(\alpha ,\beta ,k,\tilde{q}\right)$ for some k and $\tilde{q}$, and if G(α,β,q) is concave in $T_{G}\left(\alpha ,\beta ,k,\tilde{q}\right)$, then*

$$\begin{array}{cc} \; & G\left(\alpha ,\beta ,q_{*}\right)-G\left(\alpha ,\beta ,q_{N},Q_{N}\right)\leq \frac{\bar{\theta }\alpha +\theta \beta }{N}\cdot D_{UVX}(q_{*}\left|\right|q_{0}). \end{array}$$

The estimation for the one-step change in the function value for UVOB is presented in the following proposition.

**Proposition** **4.** *Given $\left(\alpha_{m},\beta_{m}\right)$, suppose that Algorithm 4 converges to the optimal variables ${\tilde{q}}_{*}$ and ${\tilde{Q}}_{*}$ such that ${\tilde{q}}_{*}=q\left[{\tilde{Q}}_{*}\right]$ and ${\tilde{Q}}_{*}=Q\left[{\tilde{q}}_{*}\right]$. Letting $\left(\alpha_{m+1},\beta_{m+1}\right)$ be updated using Equations (39) and (40) and letting $q_{0}={\tilde{q}}_{*}$ be the initial point for the next round, we have*

(43) $$\begin{array}{c} G\left(\alpha_{m+1},\beta_{m+1},q_{1},Q_{1}\right)\approx G\left(\alpha_{m},\beta_{m},{\tilde{q}}_{*}\right)-\frac{{\left(\alpha_{m+1}-\alpha_{m}\right)}^{2}}{\tau_{m}}-\frac{{\left(\beta_{m+1}-\beta_{m}\right)}^{2}}{\tau_{m}}. \end{array}$$

## 5. Numerical Results

We take the binary skew-symmetric broadcast channel p(y,z|x) as the test channel. The conditional probability matrices are

$$\begin{array}{c} P_{Y|X}=\left[\begin{array}{cc} 1 & 0\\ 0.5 & 0.5 \end{array}\right],\;P_{Z|X}=\left[\begin{array}{cc} 0.5 & 0.5\\ 0 & 1 \end{array}\right]. \end{array}$$

This is perhaps the simplest broadcast channel for which the capacity region is still unknown.

This broadcast channel plays a very important role in research on capacity bounds. It was first studied in [23] to show that the time-sharing random variable is useful for the Cover–van der Meulen inner bound [24,25]. Later, [26,27,28] demonstrated that the sum rate of the UVOB for this broadcast channel is strictly larger than that of the MIB, showing for the first time that at least one of these two bounds are suboptimal.

Our algorithms are important in at least the following sense: supposing that it is not known whether the MIB matches the UVOB (or the other two bounds for a new scenario) and we want to check this; we can perform an exhaustive search on channel matrices of size 2×2 (or of higher dimensions) to check whether they match. According to the results shown below in Section 5.5, this does not take very much time compared with generic algorithms.

In the following, we apply the algorithms to compute the value of the supporting hyperplane $\theta R_{Y}+\bar{\theta }R_{Z}$, where θ=0.4. The initial values of α and β are $\alpha_{0}=\beta_{0}=0.7$. This set of parameters is feasible for UVOB, as $\bar{\theta }\alpha +\theta \beta =0.7>\max\left\{\theta ,\bar{\theta }\right\}$.

We demonstrate the algorithms in the following aspects: (1) the maximization part; (2) the minimization part; (3) the change from the maximum part to the minimization part; (4) the superlevel set; and (5) comparison with generic non-convex algorithms.

### 5.1. Maximization Part

**Figure 1:**
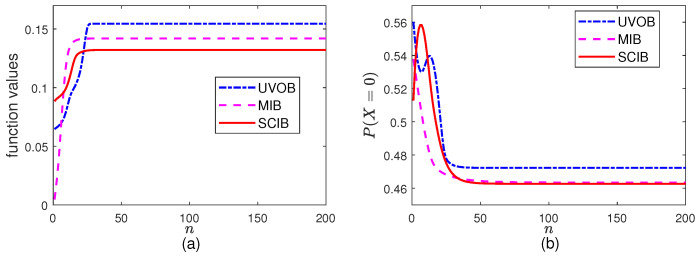
The maximization parts in the algorithms for BSSC with fixed values $\alpha_{0}=\beta_{0}=0.7$: (**a**) the objective function values and (**b**) P(X=0).

In this part, we fix α and β to the initial values and let the BA algorithms iterate for N=200 times. The results are presented in Figure 1. Because this is the maximization part, the function values increase as the iterations proceed. It is clear that the function values behave properly for fixed α and β.

### 5.2. Minimization Part

**Figure 2:**
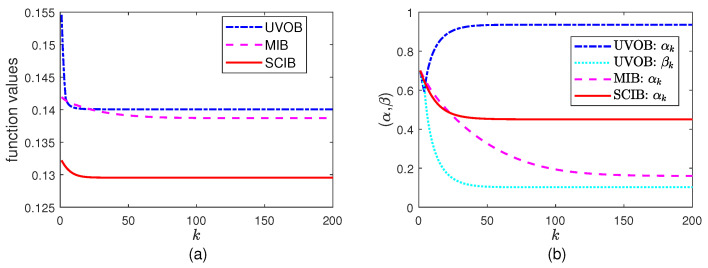
The minimization parts in the algorithms for BSSC with initial values $\alpha_{0}=\beta_{0}=0.7$: (**a**) the objective function values and (**b**) $\left(\alpha_{k},\beta_{k}\right)$.

In this part, we start with the initial $\alpha_{0}$ and $\beta_{0}$, then let the algorithms iterate for K=200 times. The results for $\left(\alpha_{k},\beta_{k}\right)$ are presented in Figure 2. Because this is the minimization part, the function values decrease as the iterations proceed. It is clear that $\alpha_{k}$ in SCIB and MIB gradually changes as *k* grows. For UVOB, it is necessary to ensure that $\bar{\theta }\alpha +\theta \beta \geq \max\left\{\theta ,\bar{\theta }\right\}$. When the updated $\left(\alpha_{k+1},\beta_{k+1}\right)$ makes $\bar{\theta }\alpha +\theta \beta$ fall below this value, it becomes necessary to scale it back. This happens approximately starting from k=5.

### 5.3. Change from Maximization to Minimization

**Figure 3:**
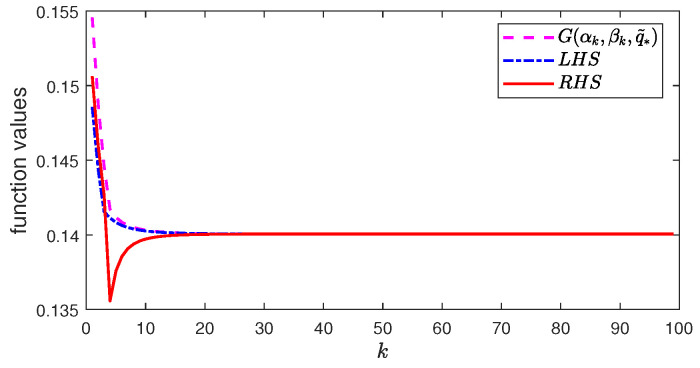
Function values of UVOB for BSSC with initial values $\alpha_{0}=\beta_{0}=0.7$.

In this part, we consider UVOB and let *K* in the algorithm be K=100. Figure 3 plots the following three values in Equation (43):

$$\begin{array}{cc} \; & G(\alpha_{k},\beta_{k},{\tilde{q}}_{*}),\\ \; & LHS:=G(\alpha_{k+1},\beta_{k+1},q_{1},Q_{1}),\\ \; & RHS:=G\left(\alpha_{k},\beta_{k},{\tilde{q}}_{*}\right)-\frac{{\left(\alpha_{k+1}-\alpha_{k}\right)}^{2}}{\tau_{k}}-\frac{{\left(\beta_{k+1}-\beta_{k}\right)}^{2}}{\tau_{k}}. \end{array}$$

As the algorithm iterates, the estimate in Equation (43) becomes more and more accurate, as exp{x}≈1+x and ln(1+x)≈x for small *x*.

### 5.4. Superlevel Set

To visualize the convergence of $q_{n}$ and its relation with the superlevel set, we take SCIB as an example and fix q(u) such that q(x|u) has two free variables. We reformulate the objective function of SCIB depicted in Equation (16) as follows:

$$\begin{array}{cc} \tilde{F}\left(\alpha ,q\left(x|u\right),Q\right)\; & =\bar{\theta }H\left(U\right)+\bar{\theta }\left(H\left(X|U\right)-H\left(X|Y,U\right)-\bar{\alpha }H\left(U|Y\right)-\alpha H\left(U|Z\right)\right)\\ \; & \;-\left(\bar{\theta }-\theta \right)\left(H\left(Y|U\right)-H\left(Y|X\right)\right)\\ \; & =\bar{\theta }H\left(U\right)+\bar{\theta }\sum_{x,u}q\left(u\right)q\left(x|u\right)\left(d\left[Q\right]\left(u,x\right)-\ln q\left(x|u\right)\right), \end{array}$$

where

$$\begin{array}{c} d\left[Q\right]\left(u,x\right)=\sum_{y,z}p\left(y,z|x\right)\left(\ln Q\left(x|y,u\right)Q{\left(u|y\right)}^{\bar{\alpha }}Q{\left(u|z\right)}^{\alpha }+\frac{\bar{\theta }-\theta }{\bar{\theta }}\ln\frac{Q(y|u)}{p(y|x)}\right). \end{array}$$

**Figure 4:**
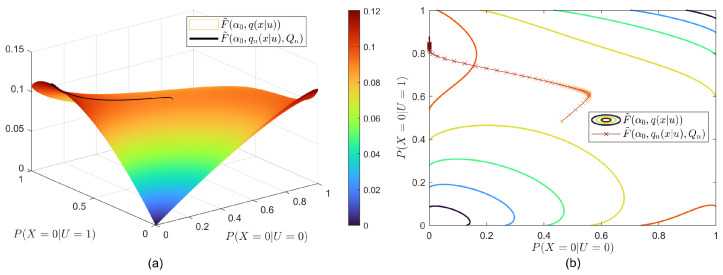
Function values of SCIB for BSSC with the initial value $\alpha_{0}=0.7$ and fixed probability vector $P_{U}=\left(0.3,0.7\right)$: (**a**) 3D view and (**b**) contour view.

In particular, we fix $\alpha_{0}=0.7$ and $P_{U}=\left(0.3,0.7\right)$, then use the algorithm to find the values of P(X=0|U=0) and P(X=0|U=1). The results are shown in Figure 4. In this case, $q_{n}$ for large enough *n* lies in the concave part of the superlevel set, meaning that the algorithm converges. Here, it should be mentioned that it is possible that the algorithm may not converge to the optimal point for some initial $q_{0}$s that do not lie in the concave part.

### 5.5. Comparison with Generic Non-Convex Algorithms

Here, we compare our algorithms with the following generic algorithms implemented using the “fmincon” MATLAB function: interior-point, active-set, and sequential quadratic programming (sqp). For simplicity, we only compare the sum rate of MIB, for which the optimal value is 0.2506717… nats (0.3616428… bits). The optimization problem for computing the sum rate is

$$\begin{array}{c} \max_{q(u,v,w,x)}\min\left\{I\left(W;Y\right),I\left(W;Z\right)\right\}+I\left(U;Y|W\right)+I\left(V;Z|W\right)-I\left(U;V|W\right). \end{array}$$

According to Lemma 3, the cardinality size is $\left|U\right|\cdot \left|V\right|\cdot \left|W\right|\cdot \left|X\right|={\left|X\right|}^{3}\left(|X|+4\right)=48$.

Notice that we do not carry out a comparison with the method in [16], as it cannot be applied to cases where there is a minimum. For scenarios in which [16] can be used, our algorithms degenerate to the method in [16].

The initial point of q(u,v,w,x) is randomly generated for all the algorithms. Table 2 lists the experimental results. For the first three algorithms, a randomly picked starting point usually does not provide a good enough result. Thus, we ran the first three algorithms multiple times until the best function value hit 0.2506 in order to test their effectiveness. It is clear from the table that only sqp can be considered comparable to our algorithms.

For further comparison with sqp, we randomly generated broadcast channels with cardinalities of |X|=3, 4, 5, 6, and |Y|=|Z|=|X|. The corresponding dimensions are ${\left|X\right|}^{3}\left(|X|+4\right)=189$, 512, 1125, 2160. Because the optimal sum rate is not yet known, we ran sqp once to record the running time. The results in Table 3 suggest that our algorithms are highly scalable. This meets our expectation, as the updating formulae in Equations (5) and (7) are all explicit and can be computed rapidly.

## 6. Discussion and Conclusions

### 6.1. Initial Points of Algorithms

Taking MIB as an example, we next discuss how to choose the initial points. When there is no prior knowledge on the optimization problem, the initial point is usually generated randomly. In this paper, Theorem 6 and Lemma 7 provide some guidance on the choice of the initial point $p_{0}\left(u,v,w,x\right)$. A possibly workable method is to randomly generate an initial point and slightly perturb it to check whether these two points satisfy the inequality in Lemma 7. If the answer is no, then it is possible that the objective function is not concave in the neighbourhood of this point, and we continue to generate new initial points.

For the initial point $\alpha_{0}$, because it lies in [0,1] it is affordable to perform a grid search, especially when |X| is small. For example, we can take 0.1 as the equal space and try each $\alpha_{0}\in \left\{0,\;0.1,\;0.2,\;\ldots ,\;1\right\}$. This approach can to some extent help us avoid becoming stuck in local extreme points.

### 6.2. J Version Outer Bound

As mentioned earlier, the best general outer bound is the J version outer bound proposed in [6]. However, the evaluation of this outer bound turns out to be even harder, as there are additional constraints on the free variables and the auxiliary channel with the joint distribution

$$\begin{array}{c} q\left(x\right)q\left(u,v,w|x\right)q\left(\tilde{u},\tilde{v},\tilde{w}|x\right)q\left(\hat{u},\hat{v},\hat{w}|x\right)p\left(y,z|x\right)T\left(j|x,y,z\right). \end{array}$$

These constraints are presented in Equations (18a)–(18c) and (19a)–(19c) in [6]. Taking Equations (18a) and (19a) as an example,

$$\begin{array}{cc} (18a):\; & I\left(\tilde{W};Z\right)-I\left(\tilde{W};J\right)+I\left(\hat{W};J\right)-I\left(\hat{W};Y\right)=I\left(W;Z\right)-I\left(W;Y\right),\\ (19a):\; & 0\leq I\left(X;Z|\tilde{U},\tilde{W}\right)-I\left(X;J|\tilde{U},\tilde{W}\right)\leq I\left(\tilde{V};Z|\tilde{W}\right)-I\left(\tilde{V};J|\tilde{W}\right). \end{array}$$

Direct application of Equation (7) does not yield an updated q[Q] guaranteed to satisfy these constraints; thus, the design of BA algorithms for the J version outer bound should carefully address this kind of problem. We leave this for future research.

Finally, to conclude our paper, the extension of the BA algorithm to inner and outer bounds for general broadcast channels encounters max–min problems. We have shown that the max–min order can be changed to min–max. Based on this observation, we have designed BA algorithms for the maximization parts and gradient descent algorithms for the minimization parts, then performed convergence analysis and numerical experiments to support our analysis. We have compared our algorithms to the following generic non-convex algorithms: interior-point, active-set, and sequential quadratic programming. The results show that our algorithms are both effective and efficient.

## Figures and Tables

**Table 1:** Comparison of typical scenarios related to the BA algorithm.

Channel	Reference	Objective	Form	Algorithm
point-to-point	[12,13]	capacity	max	BA
multiple access	[14]	sum-rate	max	BA
multiple access	[15]	inner/outer bounds	max	relaxation
degraded broadcast	[16]	capacity region	max	BA
general broadcast	this paper	inner/outer bounds	max–min	BA + gradient

**Table 2:** Comparison with generic non-convex algorithms on BSSC.

Method	Time (Seconds)	Sum-Rate of MIB (Nats)
interior-point	513.82	0.25060…
active-set	2438.57	0.25061…
sqp	1.7621	0.25067…
this paper	0.0629	0.25067…

**Table 3:** Comparison with sqp on random channels with alphabet sizes |X|=3,4,5,6.

Method	Time (Seconds)				Sum-Rate of MIB (Nats)			
	|X| = 3	|X| = 4	|X| = 5	|X| = 6	|X| = 3	|X| = 4	|X| = 5	|X| = 6
sqp	2.6342	22.44	168.12	1065.51	0.1840	0.2348	0.1983	0.2351
this paper	0.0771	0.1031	0.1450	0.2086	0.1863	0.2375	0.2019	0.2423

## Data Availability

The original contributions presented in the study are included in the article, further inquiries can be directed to the corresponding author.

## Appendix A. Proofs of Results in Section 1

**Proof** **of** **Theorem** **1.** For the first property, Fox fixed *q*, the concavity is clear, as lnQ is concave in *Q*. The equality in Equation (6) is easy to show, as if we take Q[q](x|y) to be q(x|y) then the function C(q,Q[q]) in Equation (2) reduces to C(q) in Equation (1). The maximum can be proved using the Kullback–Leibler divergence:

$$\begin{array}{cc} C(q,Q[q])-C(q,Q)\; & =C(q)-C(q,Q)\\ \; & =\sum_{x,y}q\left(x\right)p\left(y|x\right)\ln\frac{q(x|y)}{Q(x|y)}\\ \; & =\sum_{x,y}q\left(y\right)q\left(x|y\right)\ln\frac{q(x|y)}{Q(x|y)}\\ \; & =D_{X|Y}\left(q||Q\right)\\ \; & \geq 0. \end{array}$$

For the second property, we can reformulate C(q,Q) as

$$\begin{array}{c} C\left(q,Q\right)=H\left(X\right)-\sum_{x}q\left(x\right)\left(\sum_{y}p\left(y|x\right)\ln Q\left(x|y\right)\right). \end{array}$$

The concavity holds, as the first term H(X) is concave and the second term is linear. To find the maximum q[Q], because there is a constraint $\sum_{x}q\left(x\right)=1$, we consider the derivative of the Lagrangian:

$$\begin{array}{cc} 0\; & =\frac{\partial }{\partial q(x)}L\left(\lambda ,q,Q\right)\\ \; & =\frac{\partial }{\partial q(x)}\sum_{x^{\prime }}q\left(x^{\prime }\right)\left(d\left[Q\right]\left(x^{\prime }\right)-\ln q\left(x^{\prime }\right)\right)-\lambda \left(1-\sum_{x^{\prime }}q\left(x^{\prime }\right)\right)\\ \; & =d[Q](x)-\ln q(x)-1-\lambda . \end{array}$$

This implies that q(x)=exp{d[Q](x)−1−λ} for all *x*. The common term 1+λ can be eliminated by normalization, as depicted in Equation (7). We can then verify the function value as follows:

$$\begin{array}{cc} C(q[Q],Q)\; & =\sum_{x}q\left[Q\right]\left(x\right)\left(d\left[Q\right]\left(x\right)-\ln q\left[Q\right]\left(x\right)\right)\\ \; & \overset{\left(a\right)}{=}\sum_{x}q\left[Q\right]\left(x\right)\left(\ln q\left[Q\right]\left(x\right)+\ln\sum_{x^{\prime }}\exp\left\{d\left[Q\right]\left(x^{\prime }\right)\right\}-\ln q\left[Q\right]\left(x\right)\right)\\ \; & =\ln\sum_{x^{\prime }}\exp\left\{d\left[Q\right]\left(x^{\prime }\right)\right\}, \end{array}$$

where (a) is due to Equation (7). The second equality is again per Equation (7). □

**Proof** **of** **Theorem** **2.** The basic idea is to show that the sum is bounded for decreasing and positive numbers $C-C(q_{n},Q_{n})$. The monotonicity $C\left(q_{n+1},Q_{n+1}\right)\geq C\left(q_{n},Q_{n}\right)$ is clear, as we are performing alternating maximization; thus,

$$C\left(q_{n+1},Q_{n+1}\right)=C\left(q\left[Q_{n+1}\right],Q_{n+1}\right)\geq C\left(q_{n},Q_{n+1}\right)=C\left(q_{n},Q\left[q_{n}\right]\right)\geq C\left(q_{n},Q_{n}\right).$$

The positiveness of $C-C(q_{n},Q_{n})$ is because $C\geq C\left(q_{n}\right)\geq C\left(q_{n},Q_{n}\right)$. Let $q_{*}$ achieve the maximum of C(q); then,

$$\begin{array}{cc} \; & C-C\left(q_{n},Q_{n}\right)-\sum_{x}q_{*}\left(x\right)\ln\frac{q_{n}\left(x\right)}{q_{n-1}\left(x\right)}\\ \; & =C\left(q_{*},Q\left[q_{*}\right]\right)-C\left(q_{n},Q_{n}\right)-\sum_{x}q_{*}\left(x\right)\ln\frac{q_{n}\left(x\right)}{q_{n-1}\left(x\right)}\\ \; & \overset{\left(a\right)}{=}\sum_{x}q_{*}\left(x\right)\left(d\left[Q\left[q_{*}\right]\right]\left(x\right)-\ln q_{*}\left(x\right)-d\left[Q_{n}\right]\left(x\right)+\ln q_{n}\left(x\right)-\ln\frac{q_{n}\left(x\right)}{q_{n-1}\left(x\right)}\right)\\ \; & =\sum_{x}q_{*}\left(x\right)\left(d\left[Q\left[q_{*}\right]\right]\left(x\right)-\ln q_{*}\left(x\right)-d\left[Q\left[q_{n-1}\right]\right]\left(x\right)+\ln q_{n-1}\left(x\right)\right)\\ \; & \overset{\left(b\right)}{=}\sum_{x}p_{*}\left(x\right)\sum_{y}p\left(y|x\right)\left(\ln\frac{p(y|x)}{q_{*}\left(y\right)}-\ln\frac{p(y|x)}{q_{n-1}\left(y\right)}\right).\\ \; & =\sum_{x,y}q_{*}\left(x\right)p\left(y|x\right)\ln\frac{q_{n-1}\left(y\right)}{q_{*}\left(y\right)}\\ \; & =-D_{Y}(q_{*}\left|\right|q_{n-1})\\ \; & \leq 0, \end{array}$$

where (a) is due to Equation (8) and (b) holds according to Equation (9). Now, we can bound the sum as follows:

$$\begin{array}{cc} \sum_{n=1}^{N}\left(C-C\left(q_{n},Q_{n}\right)\right)\; & \leq \sum_{n=1}^{N}\sum_{x}q_{*}\left(x\right)\ln\frac{q_{n}\left(x\right)}{q_{n-1}\left(x\right)}\\ \; & =\sum_{x}q_{*}\left(x\right)\ln\frac{q_{N}\left(x\right)}{q_{0}\left(x\right)}\\ \; & =\sum_{x}q_{*}\left(x\right)\ln\frac{q_{N}\left(x\right)q_{*}\left(x\right)}{q_{0}\left(x\right)q_{*}\left(x\right)}\\ \; & =D_{X}(q_{*}\left|\right|q_{0})-D_{X}(q_{*}\left|\right|q_{N})\\ \; & \leq D_{X}(q_{*}\left|\right|q_{0}). \end{array}$$

The last term is finite, as $q_{0}>0$. This implies $C(q_{n},Q_{n})\to C$. □

## Appendix B. Proofs of Results in Section 3

**Proof** **of** **Lemma** **1.** The equivalence B=C is already stated without proof in Chapter 5.3 and Chapter 5.6 of [29]. We present the proof here for completeness. It is clear that A⊆B⊆C. Thus, we only need to prove C⊆A. It suffices to show that the corner points of C(U,X) lie inside A. Because C(U,X) is a trapezoid with one corner point (0,0), there are at most three nontrivial corner points, as follows:

1. Lower right $\left(r_{1},0\right)$, where $r_{1}=\min\left\{I\left(X;Y\right),I\left(X;Y|U\right)+I\left(U;Z\right)\right\}$. Because $r_{1}\leq I\left(X;Y\right)$, we have $\left(r_{1},0\right)\in A\left(\emptyset ,X\right)$.
2. Upper left $\left(0,r_{2}\right)$, where $r_{2}=\min\left\{I\left(U;Z\right),I\left(X;Y\right)\right\}$. Clearly, $\left(0,r_{2}\right)\in A\left(X,X\right)$.
3. Upper right (min{I(X;Y|U),I(X;Y)−I(U;Z)},I(U;Z)); this corner point exists when I(U;Z)≤I(X;Y). We can consider two cases:
  (a) I(U;Y)≥I(U;Z): the corner point (I(X;Y|U),I(U;Z)) is inside A(U,X).
  (b) I(U;Y)≤I(U;Z): the corner point is (I(X;Y)−I(U;Z),I(U;Z)). It suffices to consider I(U;Y)≤I(U;Z)≤I(X;Y), as otherwise this point does not exist. Now, let α be such that (1−α)I(X;Y)+αI(U;Y)=I(U;Z). We can construct a pair $\left(\hat{U},\hat{X}\right)$ such that this corner point is inside $A(\hat{U},\hat{X})$. Considering the random variables Q∼Bernoulli(α), $\tilde{U}=X$ when Q=0 and $\tilde{U}=U$ when Q=1, we can let $\hat{U}=\left(\tilde{U},Q\right)$, $\hat{X}=X$; then, $I\left(\hat{X};Y|\hat{U}\right)=0+\alpha I\left(X;Y|U\right)=I\left(X;Y\right)-I\left(U;Z\right)$. Now, we have $I\left(\hat{U};Z\right)\geq I\left(\tilde{U};Z|Q\right)\geq I\left(U;Z\right)$ and $I\left(\hat{U};Y\right)\geq I\left(\tilde{U};Y|Q\right)=I\left(U;Z\right)$; hence, $\min\left\{I\left(\hat{U};Z\right),I\left(\hat{U};Y\right)\right\}\geq I\left(U;Z\right)$.

The proof is finished. □

**Proof** **of** **Theorem** **3.** We can use A(U,X) in Lemma 1 to compute the supporting hyperplanes. When $\theta \in [\frac{1}{2},1]$, then $\theta R_{Y}+\bar{\theta }R_{Z}$ is bounded from above by

$$\begin{array}{cc} \max_{q(u,x)}\theta I\left(X;Y|U\right)+\bar{\theta }I\left(U;Y\right)\; & =\max_{q(u,x)}\theta \left(I\left(X;Y\right)-I\left(U;Y\right)\right)+\bar{\theta }I\left(U;Y\right)\\ \; & =\max_{q(u,x)}\theta I\left(X;Y\right)+\left(1-2\theta \right)I\left(U;Y\right)\\ \; & \leq \max_{q(x)}\theta I\left(X;Y\right). \end{array}$$

On the other hand, this upper bound is achieved by setting U=∅ in A(U,X). When $\theta \in [0,\frac{1}{2})$, we first have

$$\begin{array}{c} F=\max_{p(u,x)}\min_{\alpha \in [0,1]}\theta I\left(X;Y|U\right)+\bar{\theta }\alpha I\left(U;Z\right)+\bar{\theta }\bar{\alpha }I\left(U;Y\right). \end{array}$$

To show that the maximum and minimum can be exchanged, we use Lemma 2; in particular, letting d=2, $\lambda_{1}=\alpha$, and $\lambda_{2}=\bar{\alpha }$, we have

$$\begin{array}{cc} T_{1}\left(q\left(u,x\right)\right) & =\theta I\left(X;Y|U\right)+\bar{\theta }I\left(U;Z\right),\\ T_{2}\left(q\left(u,x\right)\right) & =\theta I\left(X;Y|U\right)+\bar{\theta }I\left(U;Y\right). \end{array}$$

Then, the objective function F(α,q) equals $\lambda_{1}T_{1}+\lambda_{2}T_{2}$. It remains to prove that T is convex. Assuming that $(a_{1},a_{2})\in T$ for some $q_{a}\left(u,x\right)$ and that $(b_{1},b_{2})\in T$ for some $q_{b}\left(u,x\right)$ while letting $\tilde{U}=\left(U,Q\right)$, where Q∼Bernoulli(β) and $\left(U,X\right)∼q_{a}\left(u,x\right)$ if Q=1 and $\left(U,X\right)∼q_{b}\left(u,x\right)$ if Q=0, we then have

$$\begin{array}{cc} T_{1}\left(\beta q_{a}+\bar{\beta }q_{b}\right)\; & =T_{1}\left(q\left(\tilde{u},x\right)\right)\\ \; & =\theta I\left(X;Y|U,Q\right)+\bar{\theta }I\left(U,Q;Z\right)\\ \; & \geq \theta I\left(X;Y|U,Q\right)+\bar{\theta }I\left(U;Z|Q\right)\\ \; & =\beta T_{1}\left(q_{a}\right)+\bar{\beta }T_{1}\left(q_{b}\right). \end{array}$$

Similar inequalities hold for $T_{2}$. This proves the convexity, and hence the exchange. Now, we can show that the expression of *F* can be simplified for $\theta \in [0,\frac{1}{2})$ and $\alpha \leq \frac{1-2\theta }{1-\theta }$. Noting that I(U;Y)=I(X;Y)−I(X;Y|U) and I(U;Z)=I(X;Z)−I(X;Z|U), we have

$$\begin{array}{cc} \; & \theta I\left(X;Y|U\right)+\bar{\theta }\bar{\alpha }I\left(U;Y\right)+\bar{\theta }\alpha I\left(U;Z\right)\\ \; & =\bar{\theta }\bar{\alpha }I\left(X;Y\right)+\bar{\theta }\alpha I\left(X;Z\right)+\left(\theta -\bar{\theta }\bar{\alpha }\right)I\left(X;Y|U\right)-\bar{\theta }\alpha I\left(X;Z|U\right). \end{array}$$

When $\alpha \leq \frac{1-2\theta }{1-\theta }$, i.e., $\theta -\bar{\theta }\bar{\alpha }\leq 0$, the last two terms above have a non-positive sum. The maximum value equals zero, and can be achieved by taking U=X. This finishes the proof of the expression. The cardinality can be proved as follows:

$$\begin{array}{cc} \; & \theta I\left(X;Y|U\right)+\bar{\theta }\bar{\alpha }I\left(U;Y\right)+\bar{\theta }\alpha I\left(U;Z\right)\\ \; & =\bar{\theta }\bar{\alpha }I\left(X;Y\right)+\bar{\theta }\alpha I\left(X;Z\right)+\left(\theta -\bar{\theta }\bar{\alpha }\right)I\left(X;Y|U\right)-\bar{\theta }\alpha I\left(X;Z|U\right)\\ \; & =f\left(q\left(x\right)\right)+\sum_{u}q\left(u\right)g\left(q\left(x|u\right)\right), \end{array}$$

where *f* and *g* are some continuous functions corresponding to the mutual information. Subject to fixed marginal q(x), the maximum of $\sum_{u}q\left(u\right)g\left(q\left(x|u\right)\right)$ over all feasible q(u) and q(x|u) is the upper concave envelope of the function *g* evaluated at q(x). Notice that as the degree of freedom of the distribution q(x) is |X|−1, it suffices to consider |U|≤X|−1+1=|X| for evaluating the envelope. □

**Proof** **of** **Theorem** **4.** For fixed q(u,v,x), in the pentagon of the UVOB there are at most two corner points in the first quadrant, namely, the upper left and lower right ones. The line connecting these two points has slope −1. We need to compute the the supporting hyperplane value $G=\max_{(R_{Y},R_{Z})\in UVOB}\theta R_{Y}+\bar{\theta }R_{Z}$, where θ∈(0,1). For the case $\theta \in (0,\frac{1}{2}]$, note that the slope of the line $\theta R_{Y}+\bar{\theta }R_{Z}=G$ is $-\theta /\bar{\theta }\geq -1$; thus, it suffices to consider the upper left corner point. The expression of this point is different when q(u,v,x) falls into one of the following two sets:

$$\begin{array}{cc} S_{1} & =\{q(u,v,x):I(V;Z)\leq I(U;Y)+I(X;Z|U)\},\\ S_{2} & =\{q(u,v,x):I(V;Z)\geq I(U;Y)+I(X;Z|U)\}. \end{array}$$

When $q\in S_{1}$, this corner point and the corresponding expression of $l_{1}\left(q\right):=\theta R_{Y}+\bar{\theta }R_{Z}$ are

$$\begin{array}{cc} \; & \left(\min\{I(U;Y),I(U;Y)+I(X;Z|U)-I(V;Z),I(X;Y|V)\},\;I(V;Z)\right),\\ l_{1}\left(q\right)=\; & \theta \cdot \min\left\{I\left(U;Y\right),I\left(U;Y\right)+I\left(X;Z|U\right)-I\left(V;Z\right),I\left(X;Y|V\right)\right\}+\bar{\theta }I\left(V;Z\right). \end{array}$$

Otherwise, when $q\in S_{2}$, this corner point and corresponding expression of $l_{2}\left(q\right):=\theta R_{Y}+\bar{\theta }R_{Z}$ are

$$\begin{array}{cc} \; & \left(0,\;I(U;Y)+I(X;Z|U)\right),\\ l_{2}\left(q\right)=\; & \bar{\theta }\left(I(U;Y)+I(X;Z|U)\right). \end{array}$$

Now, the supporting hyperplane value is

$$\begin{array}{c} G=\max\{\max_{q\in S_{1}}l_{1}\left(q\right),\;\max_{q\in S_{2}}l_{2}\left(q\right)\}. \end{array}$$

We want to show that

$$\begin{array}{c} \max_{q\in S_{2}}l_{2}\left(q\right)=\max_{q\in S_{2}}l_{1}\left(q\right). \end{array}$$

For the left hand-side term, we have

$$\begin{array}{cc} \max_{q\in S_{2}}l_{2}\left(q\right)\; & =\max_{q\in S_{2}}\bar{\theta }\left(I(U;Y)+I(X;Z|U)\right)\\ \; & \leq \max_{q\in S_{2}}\bar{\theta }I\left(V;Z\right)\\ \; & \leq \max_{q\in S_{2}}\bar{\theta }I\left(X;Z\right), \end{array}$$

where the equalities hold with U=∅, V=X and this choice is in $S_{2}$. For the other term,

$$\begin{array}{cc} \max_{q\in S_{2}}l_{1}\left(q\right)\; & =\max_{q\in S_{2}}\theta \left(I\left(U;Y\right)+I\left(X;Z|U\right)-I\left(V;Z\right)\right)+\bar{\theta }I\left(V;Z\right)\\ \; & \leq \max_{q\in S_{2}}\bar{\theta }I\left(V;Z\right)\\ \; & \leq \max_{q\in S_{2}}\bar{\theta }I\left(X;Z\right), \end{array}$$

where the equalities hold using the same settings as above. Hence, we have the supporting hyperplane value

$$\begin{array}{c} G=\max\left\{\max_{q\in S_{1}}l_{1}\left(q\right),\;\max_{q\in S_{2}}l_{1}\left(q\right)\right\}=\max_{q}l_{1}\left(q\right). \end{array}$$

We can simplify this expression as follows:

$$\begin{array}{cc} \max_{q}l_{1}\left(q\right)=\max_{q}\min_{a,b\in [0,1]} & \;\theta \left(1-a\right)I\left(U;Y\right)+\theta a\left(1-b\right)\left(I(U;Y)+I(X;Z|U)-I(V;Z)\right)\\ \; & +\theta abI\left(X;Y|V\right)+\bar{\theta }I\left(V;Z\right)\\ =\max_{q}\min_{a,b\in [0,1]} & \;\theta (1-ab)I(U;Y)+\theta a(1-b)I(X;Z|U)\\ \; & +(\bar{\theta }-\theta a\left(1-b\right))I\left(V;Z\right)+\theta abI\left(X;Y|V\right). \end{array}$$

Letting $\alpha =1-\theta a\left(1-b\right)/\bar{\theta }$, β=1−ab, we have α,β∈[0,1], $\bar{\theta }\alpha +\theta \beta =\bar{\theta }+\theta \left(1-a\right)$, and the supporting hyperplane value is

$$\begin{array}{cc} \theta R_{Y}+\bar{\theta }R_{Z}=\max_{q}\min_{\alpha ,\beta \in [0,1]} & \;\bar{\theta }\alpha I\left(V;Z\right)+\bar{\theta }\bar{\alpha }I\left(X;Z|U\right)+\theta \beta I\left(U;Y\right)+\theta \bar{\beta }I\left(X;Y|V\right). \end{array}$$

Notice that the range of $\bar{\theta }\alpha +\theta \beta$ is $\left[\max\left\{\theta ,\bar{\theta }\right\},1\right]$. Within this range, the reverse mapping from (α,β) to (a,b) is

$$\begin{array}{c} a=\frac{1-(\bar{\theta }\alpha +\theta \beta )}{\theta },\;b=\frac{\theta \bar{\beta }}{\theta \bar{\beta }+\bar{\theta }\bar{\alpha }}. \end{array}$$

Notice that when $\theta \in [\frac{1}{2},1)$, we have $\bar{\theta }\in \left(0,\frac{1}{2}\right]$; we can use similar reasoning as above (by swapping *Y* and *Z*, $R_{Y}$ and $R_{Z}$, *U* and *V*, θ and $\bar{\theta }$, and finally α and β) to obtain

$$\begin{array}{cc} \bar{\theta }R_{Z}+\theta R_{Y}=\max_{q}\min_{\alpha ,\beta \in [0,1]} & \;\theta \beta I\left(U;Y\right)+\theta \bar{\beta }I\left(X;Y|V\right)+\bar{\theta }\alpha I\left(V;Z\right)+\bar{\theta }\bar{\alpha }I\left(X;Z|U\right). \end{array}$$

The constraint then becomes $\theta \beta +\bar{\theta }\alpha =\theta +\bar{\theta }\left(1-a\right)$, for which the range is again $\left[\max\left\{\bar{\theta },\theta \right\},1\right]$. Thus, the expression and the constraints are the same as for the case where $\theta \in (0,\frac{1}{2}]$. Putting these two cases together, we have the characterization of the supporting hyperplanes. To exchange the max–min, we again use Lemma 2. The proof is similar to that of Theorem 3, and as such we omit the details, providing only the setting for $\theta \in (0,\frac{1}{2}]$, where $G=\max_{q}l_{1}\left(q\right)$: d=3 and the functions are

$$\begin{array}{cc} T_{1}\left(q\right) & =\theta I\left(U;Y\right)+\bar{\theta }I\left(V;Z\right),\\ T_{2}\left(q\right) & =\theta \left(I\left(U;Y\right)+I\left(X;Z|U\right)-I\left(V;Z\right)\right)+\bar{\theta }I\left(V;Z\right),\\ T_{3}\left(q\right) & =\theta I\left(X;Y|V\right)+\bar{\theta }I\left(V;Z\right). \end{array}$$

The proof of the cardinality bounds is similar to that of Theorem 3. □

## Appendix C. Proofs of Results in Section 4

**Proof** **of** **Lemma** **5.** Let *A* represent a generic random variable. The gradient of H(A) can be calculated as follows:

$$\begin{array}{cc} \frac{\partial H(A)}{\partial q(u,x)}\; & =\frac{\partial }{\partial q(u,x)}\left(-\sum_{a}q\left(a\right)\ln q\left(a\right)\right)\\ \; & =-\sum_{a}\frac{\partial q(a)}{\partial q(u,x)}\ln q\left(a\right)-\sum_{a}q\left(a\right)\frac{1}{q(a)}\frac{\partial q(a)}{\partial q(u,x)}\\ \; & =-\sum_{a}\frac{\partial q(a)}{\partial q(u,x)}\left(1+\ln q\left(a\right)\right). \end{array}$$

For the particular term H(U|Y) in F(α,q), the gradient is

$$\begin{array}{cc} \frac{\partial \left(H(U,Y)-H(Y)\right)}{\partial q(u,x)}\; & =-\sum_{u^{\prime },y}\frac{\partial q(u^{\prime },y)}{\partial q(u,x)}\left(1+\ln q\left(u^{\prime },y\right)\right)+\sum_{y}\frac{\partial q(y)}{\partial q(u,x)}\left(1+\ln q\left(y\right)\right)\\ \; & =-\sum_{y}p\left(y|x\right)\left(1+\ln q\left(u,y\right)\right)+\sum_{y}p\left(y|x\right)\left(1+\ln q\left(y\right)\right)\\ \; & =-\sum_{y}p\left(y|x\right)\ln q\left(u|y\right). \end{array}$$

Now, we can calculate the first-order term in Equation (41):

$$\begin{array}{cc} -{\left(q_{2}-q_{1}\right)}^{T}\nabla H_{q_{1}}\left(U|Y\right)\; & =\sum_{u,x,y}\left(q_{2}\left(u,x\right)-q_{1}\left(u,x\right)\right)p\left(y|x\right)\ln q_{1}\left(u|y\right)\\ \; & =\sum_{u,y}q_{2}\left(u,y\right)\ln q_{1}\left(u|y\right)+H_{q_{1}}\left(U|Y\right). \end{array}$$

Finally, the left-hand side of Equation (41) equals

$$\begin{array}{cc} H_{q_{2}}\left(U|Y\right)-H_{q_{1}}\left(U|Y\right)-{\left(q_{2}-q_{1}\right)}^{T}\nabla H_{q_{1}}\left(U|Y\right)\; & =H_{q_{2}}\left(U|Y\right)+\sum_{u,y}q_{2}\left(u,y\right)\ln q_{1}\left(u|y\right)\\ \; & =-D_{U|Y}(q_{2}\left|\right|q_{1}). \end{array}$$

For the other terms in Equation (16), we can perform similar calculations to obtain the desired inequality. □

**Proof** **of** **Lemma** **6.** Consider the superlevel set ${\tilde{S}}_{F}\left(\alpha ,k\right)$ of the function $F(\alpha ,q,Q\left[q_{n}\right])$. According to Equation (6), $F\left(\alpha ,q,Q\left[q_{n}\right]\right)\leq F\left(\alpha ,q\right)$ for all *q*; thus, ${\tilde{S}}_{F}\left(\alpha ,k\right)\subseteq S_{F}\left(\alpha ,k\right)$. From Equation (6), $F\left(\alpha ,q_{n},Q\left[q_{n}\right]\right)=F\left(\alpha ,q_{n}\right)$, we have $q_{n}\in {\tilde{S}}_{F}\left(\alpha ,k\right)$; further, because $F(\alpha ,q,Q\left[q_{n}\right])$ is concave in *q*, ${\tilde{S}}_{F}\left(\alpha ,k\right)$ is a convex set, and as such is connected. This implies that ${\tilde{S}}_{F}\left(\alpha ,k\right)\subseteq T_{F}\left(\alpha ,k,q_{n}\right)$. Because $q_{n+1}$ makes the function value $F(\alpha ,q,Q\left[q_{n}\right])$ larger than that of $q_{n}$, it must lie in ${\tilde{S}}_{F}\left(\alpha ,k\right)$, and as such in $T_{F}\left(\alpha ,k,q_{n}\right)$. □

**Proof** **of** **Theorem** **5.** The proof is similar to that of Theorem 2. We perform the following manipulations:

$$\begin{array}{cc} \; & F\left(\alpha ,q_{*}\right)-F\left(\alpha ,q_{n},Q_{n}\right)-\bar{\theta }\sum_{u,x}q_{*}\left(u,x\right)\ln\frac{q_{n}\left(u,x\right)}{q_{n-1}\left(u,x\right)}\\ \; & =F\left(\alpha ,q_{*},Q\left[q_{*}\right]\right)-F\left(\alpha ,q_{n},Q_{n}\right)-\bar{\theta }\sum_{u,x}q_{*}\left(u,x\right)\ln\frac{q_{n}\left(u,x\right)}{q_{n-1}\left(u,x\right)}\\ \; & \overset{\left(a\right)}{=}\bar{\theta }\sum_{u,x}q_{*}\left(u,x\right)\left(d\left[Q\left[q_{*}\right]\right]-\ln q_{*}-d\left[Q_{n}\right]+\ln q_{n}-\ln\frac{q_{n}}{q_{n-1}}\right)\;\;\;\;\;\; \end{array}$$

$$\begin{array}{cc} \; & =\bar{\theta }\sum_{u,x}q_{*}\left(u,x\right)\left(d\left[Q\left[q_{*}\right]\right]-\ln q_{*}-d\left[Q\left[q_{n-1}\right]\right]+\ln q_{n-1}\right)\\ \; & \overset{\left(b\right)}{=}\bar{\theta }(-D_{UX}(q_{*}\left|\right|q_{n-1})+D_{X|YU}+\bar{\alpha }D_{U|Y}+\alpha D_{U|Z})+\left(\bar{\theta }-\theta \right)D_{Y|U}\\ \; & \overset{\left(c\right)}{\leq }0, \end{array}$$

where (a) holds from Equation (8), (b) is due to

$$\begin{array}{cc} \; & \;\;\sum_{u,x}q_{*}\left(d\left[Q\left[q_{*}\right]\right]-d\left[Q\left[q_{n-1}\right]\right]\right)\\ \; & \;\;=\sum_{u,x,y,z}q_{*}\left(u,x\right)p\left(y,z|x\right)(\ln\frac{q_{*}\left(x|y,u\right)}{q_{n-1}\left(x|y,u\right)}{\left(\frac{q_{*}\left(u|y\right)}{q_{n-1}\left(u|y\right)}\right)}^{\bar{\alpha }}{\left(\frac{q_{*}\left(u|z\right)}{q_{n-1}\left(u|z\right)}\right)}^{\alpha }\\ \; & \;\;\;\;\;\;+\frac{\bar{\theta }-\theta }{\bar{\theta }}\ln\frac{q_{*}\left(y|u\right)}{q_{n-1}\left(y|u\right)})\\ \; & \;\;=D_{X|YU}(q_{*}\left|\right|q_{n-1})+\bar{\alpha }D_{U|Y}+\alpha D_{U|Z}+\frac{\bar{\theta }-\theta }{\bar{\theta }}D_{Y|U}, \end{array}$$

and (c) is from Lemma 5. This implies that

$$\begin{array}{cc} \sum_{n=1}^{N}\left(F\left(\alpha ,q_{*}\right)-F\left(\alpha ,q_{n},Q_{n}\right)\right)\; & \leq \bar{\theta }\sum_{u,x}q_{*}\left(u,x\right)\ln\frac{q_{N}\left(u,x\right)}{q_{0}\left(u,x\right)}\\ \; & =\bar{\theta }\sum_{u,x}q_{*}\left(u,x\right)\ln\frac{q_{N}\left(u,x\right)q_{*}\left(u,x\right)}{q_{0}\left(u,x\right)q_{*}\left(u,x\right)}\\ \; & =\bar{\theta }(D_{UX}(q_{*}\left|\right|q_{0})-D_{UX}(q_{*}\left|\right|q_{N}))\\ \; & \leq \bar{\theta }D_{UX}(q_{*}\left|\right|q_{0}). \end{array}$$

The last term is finite and positive, as $q_{0}>0$. Finally, a sequence of positive terms has a finite sum, which implies that the terms converge to zero, i.e., $F\left(\alpha ,q_{n},Q_{n}\right)\to F\left(\alpha ,q_{*}\right)$. □

**Proof** **of** **Proposition** **2.** Let $C_{m}$ and $\Delta_{d}\left(u,x\right)$ be

$$\begin{array}{cc} C_{m} & =\sum_{u,x}\exp\left\{d_{m}\left[{\tilde{Q}}_{*}\right]\left(u,x\right)\right\},\\ \Delta_{d}\left(u,x\right) & =d_{m+1}\left[{\tilde{Q}}_{*}\right]-d_{m}\left[{\tilde{Q}}_{*}\right], \end{array}$$

where $d_{k}\left[Q\right]$ is as in Equation (18) with $\alpha =\alpha_{k}$. According to Equations (7) and (8),

$$\begin{array}{cc} q_{0}={\tilde{q}}_{*}=\frac{\exp\{d_{m}\left[{\tilde{Q}}_{*}\right]\}}{\sum \exp\{d_{m}\left[{\tilde{Q}}_{*}\right]\}} & =\frac{\exp\{d_{m}\left[{\tilde{Q}}_{*}\right]\}}{C_{m}},\\ F(\alpha_{m},{\tilde{q}}_{*}) & =\bar{\theta }\ln C_{m}. \end{array}$$

Noting that $Q_{1}=Q\left[q_{0}\right]=Q\left[{\tilde{q}}_{*}\right]={\tilde{Q}}_{*}$, we estimate the difference as follows:

$$\begin{array}{cc} F\left(\alpha_{m+1},q_{1},Q_{1}\right)-F\left(\alpha_{m},{\tilde{q}}_{*}\right)\; & \overset{\left(a\right)}{=}\bar{\theta }\ln\sum \exp\left\{d_{m+1}\left[{\tilde{Q}}_{*}\right]\right\}-\bar{\theta }\ln C_{m}\\ \; & =\bar{\theta }\ln\sum \exp\left\{d_{m}\left[{\tilde{Q}}_{*}\right]\right\}\exp\left\{\Delta_{d}\right\}-\bar{\theta }\ln C_{m}\\ \; & =\bar{\theta }\ln\sum C_{m}{\tilde{q}}_{*}\exp\left\{\Delta_{d}\right\}-\bar{\theta }\ln C_{m}\\ \; & =\bar{\theta }\ln\sum {\tilde{q}}_{*}\exp\left\{\Delta_{d}\right\}\\ \; & \approx \bar{\theta }\ln\sum {\tilde{q}}_{*}\left(1+\Delta_{d}\right)\\ \; & =\bar{\theta }\ln\left(1+\sum {\tilde{q}}_{*}\Delta_{d}\right)\\ \; & \approx \bar{\theta }\sum {\tilde{q}}_{*}\Delta_{d}, \end{array}$$

where (a) holds from Equation (8) and from the fact that $Q_{1}={\tilde{Q}}_{*}$. According to the definition of d[Q] in Equation (19),

$$\begin{array}{c} \Delta_{d}=\left(\alpha_{m+1}-\alpha_{m}\right)\sum_{y,z}p\left(y,z|x\right)\ln\frac{{\tilde{Q}}_{*}\left(u|z\right)}{{\tilde{Q}}_{*}\left(u|y\right)}. \end{array}$$

The expectation of this difference is

$$\begin{array}{cc} \sum_{u,x}{\tilde{q}}_{*}\Delta_{d}\; & =\left(\alpha_{m+1}-\alpha_{m}\right)\left(I\left(U;Z\right)-I\left(U;Y\right)\right)\\ \; & \overset{\left(b\right)}{=}\left(\alpha_{m+1}-\alpha_{m}\right)\left(\alpha_{m+1}-\alpha_{m}\right)\frac{1}{-\tau_{m}\bar{\theta }}\\ \; & =-\frac{{\left(\alpha_{m+1}-\alpha_{m}\right)}^{2}}{\tau_{m}\bar{\theta }}, \end{array}$$

where (b) is due to Equation (22). The proof is finished. □
